# Model selection reveals control of cold signalling by evening-phased components of the plant circadian clock

**DOI:** 10.1111/tpj.12303

**Published:** 2013-08-05

**Authors:** Jack Keily, Dana R MacGregor, Robert W Smith, Andrew J Millar, Karen J Halliday, Steven Penfield

**Affiliations:** 1 Biosciences, College of Life and Environmental Sciences, University of ExeterStocker Road, Exeter, EX4 4QD, UK; 2 Department of Biological Sciences, University of EdinburghCH Waddington Building, Mayfield Road, Edinburgh, EH9 3JD, UK

**Keywords:** Arabidopsis, circadian clock, cold acclimation, temperature, gene network model

## Abstract

Circadian clocks confer advantages by restricting biological processes to certain times of day through the control of specific phased outputs. Control of temperature signalling is an important function of the plant oscillator, but the architecture of the gene network controlling cold signalling by the clock is not well understood. Here we use a model ensemble fitted to time-series data and a corrected Akaike Information Criterion (AICc) analysis to extend a dynamic model to include the control of the key cold-regulated transcription factors *C-REPEAT BINDING FACTOR*s *1–3* (*CBF1*, *CBF2*, *CBF3*). AICc was combined with *in silico* analysis of genetic perturbations in the model ensemble, and selected a model that predicted mutant phenotypes and connections between evening-phased circadian clock components and *CBF3* transcriptional control, but these connections were not shared by *CBF1* and *CBF2*. In addition, our model predicted the correct gating of *CBF* transcription by cold only when the cold signal originated from the clock mechanism itself, suggesting that the clock has an important role in temperature signal transduction. Our data shows that model selection could be a useful method for the expansion of gene network models.

## Introduction

Circadian clocks provide organisms with competitive advantages through the generation of rhythmic expression of key regulatory genes that time the peak of activity of essential functions. The endogenous circadian rhythm can be entrained to the external environment to enable organisms to predict daily changes and initiate responses. Central to the study of circadian clocks is not only understanding the architecture of the oscillators themselves, but also the way in which the clock affects output pathways that confer survival advantages, the maintenance of output phase and the integration of timing information into signalling pathways (Farré and Weise, 2012). A common feature of the initiation of circadian outputs is the co-option of DNA-binding transcription factors within the transcription–translation feedback loops that characterise eukaryotic circadian systems for the regulation of gene expression not associated with the oscillator itself (Suárez-López *et al*., 2001; Mikkelsen and Thomashow, 2009; Nusinow *et al.*, 2011).

**Figure 1:**
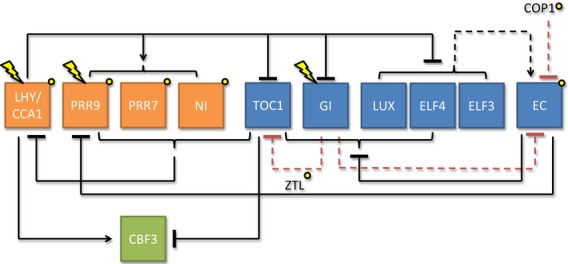
The architecture of the Arabidopsis circadian clock model used in this study (Pokhilko *et al.*, 2012) and proposed new connections to *CBF* mRNA transcription.

The Arabidopsis clock is characterised by a series of interlocking transcription/translation feedback loops (Figure 1) in which two morning-expressed and partially redundant transcription factors, LATE ELONGATED HYPOCOTYL (LHY) AND CIRCADIAN CLOCK-ASSOCIATED 1 (CCA1) repress the expression of a series of evening-expressed components, most importantly TIMING OF CAB EXPRESSION1 (TOC1) and the Evening Complex consisting of LUX ARRYTHMO (LUX), EARLY FLOWERING 3 and EARLY FLOWERING 4 (ELF3 and ELF4; Figure 1). The complexity of the plant oscillator means that our state-of-the-art understanding of clock architecture employs dynamic ordinary differential equation approaches to generate and test hypotheses (Locke *et al.*, 2006; Pokhilko *et al.*, 2010, 2012). Arabidopsis clock models have been successfully linked to output genes to generate and test hypotheses for photoperiodic signal generation (Salazar *et al.*, 2009; Song *et al.*, 2012).

Given the increasingly sophisticated understanding of gene networks underlying circadian function, a key bottleneck is understanding how to most effectively identify the mechanisms through which circadian outputs are produced. An important function of the Arabidopsis circadian clock is the regulation of temperature-responsive gene expression. The cold-induced expression of three redundant transcription factors known as *C-REPEAT BINDING FACTOR*s (*CBF*s; also known as *DREB1*; Liu *et al.*, 1998) is a key step in plant responses to cold. *CBF*s elicit tolerance to freezing temperatures by initiating a signal transduction cascade culminating in the transcription of *COR* genes that encode proteins that enhance freezing survival (Stockinger *et al.*, 1997). The ability to survive freezing is altered in plants with compromised circadian clocks, with *lhy cca1* double mutants showing increased sensitivity to cold, whereas *prr5 prr7 prr9* triple mutants show a strong enhanced ability to withstand freezing temperatures (Nakamichi *et al.*, 2009; Espinoza *et al.*, 2010; Dong *et al.*, 2011). *CBF* expression is also under circadian regulation with peak expression occurring 7–8 h after subjective dawn (Harmer *et al.*, 2000), even though maximum cold-induced expression of *CBF*s occurs approximately 4 h earlier (Fowler *et al.*, 2005). The reason for circadian regulation of the pathway is not clear, but a recent study suggests that temperature information mediated by the alternative splicing of *CCA1* can be transduced to promote freezing tolerance (Seo *et al.*, 2012). It is currently unclear how important this temperature signalling is compared with other known regulators of *CBF*s, such as INDUCER OF CBF EXPRESSION 1 (ICE1).

Several possible mechanisms for the circadian control of *CBF* expression have been proposed, including direct activation by LHY and CCA1 (Espinoza *et al.*, 2010; Dong *et al.*, 2011) and inhibition by PRR5, PRR7 and PRR9 (Nakamichi *et al.*, 2009). Of the proposed modes of regulation, only CCA1 has been shown to bind the *CBF* promoters, and the data indicate that that this interaction is direct (Dong *et al.*, 2011). In this study we use dynamic modelling to extend existing models of the Arabidopsis circadian clock (Pokhilko *et al.*, 2012) to predict the nature of the molecular connections through which *CBF*s are regulated by the circadian clock. Using dynamic simulation and application of a modified corrected Akaike Information Criterion (AICc; Akaike, 1974; Hurvich and Tsai, 1989) model selection algorithm we compare various possible models of circadian control of *CBF3* expression by optimised fitting of model parameters to time-series data. We show that AICc selects a model which not only recapitulates published data on *CBF3* expression in Arabidopsis circadian clock mutants, but also predicts a previously unknown direct regulation by TOC1 and the Evening Complex. We show that this dual regulation of *CBF*s by CCA1 and evening-phased genes predicts the known circadian gating of the cold-regulation of *CBF*s if the cold signal comes from CCA1 activation (Seo *et al.*, 2012) but not if the cold signal is external to the clock mechanism, suggesting that the circadian clock has an important role in temperature signal transduction.

## Results

### Construction of models describing the regulation of *CBF3* expression by the Arabidopsis circadian oscillator

**Figure 2:**
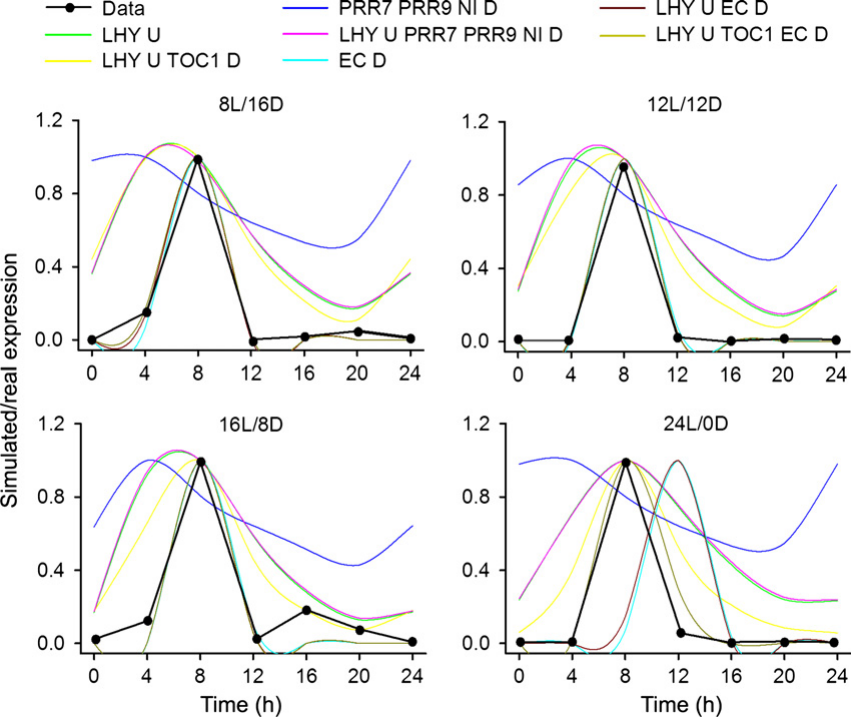
Simulation of the best model fit for each variant (coloured lines) against experimentally determined *CBF3* expression (black line) in four indicated light regimes, either short days (8L/16D), 12 h light, 12 h dark (12L/12D), long days (16L/8D) or constant light 24L/0D. U indicated *CBF3* transcription up-regulated by variable, D indicates *CBF3* transcription down-regulated by variable.

The most current Arabidopsis clock model contains representations of nine gene products that act in a multiple loop structure to control circadian period (Pokhilko *et al.*, 2012; Figure 1, and hereafter referred to as the P2012 model). Under ambient conditions three *CBF* genes (1–3) are expressed daily with a phase of peak expression 8 h after dawn, a phase which is robust against variation of the photoperiod (Mockler *et al.*, 2007; Figure 2). For simplicity we chose to model the control of *CBF3* expression because *CBF3* has the most robust expression under ambient temperatures. In the P2012 model the morning-expressed *LHY* and *CCA1* transcription factors are represented by a single variable LHY. CCA1 has been shown previously to bind the *CBF* promoters, but LHY was also found to be necessary for normal *CBF* expression (Dong *et al.*, 2011). Because this work showed that in the absence of LHY and CCA1 *CBF* expression was greatly reduced, we focussed on models that included a link promoting *CBF* expression by LHY. In addition to the simple model in which *CBF3* transcription was activated by LHY/CCA1 alone, we constructed a range of models including activation and repression of expression by various combinations of clock components (Table 1; see Supplementary Information for methods, Figures S1–S3 and Tables S1–S3). We focussed on model containing direct links to PRR proteins, as *prr5 prr7 prr9* triple mutants have been shown to have highly up-regulated *CBF* expression (Nakamichi *et al.*, 2009). Although PRR5 is not explicitly represented as a variable in P2012, variable *NI* has an expression pattern and function consistent with PRR5 and was used as a proxy. While we could not construct every conceivable model, we analysed a total of 13 variants (Table 1). We fixed parameters in the P2012 model at their published values and represented the control of *CBF3* expression with Hill functions for the simulation of transcription and exponential degradation kinetics (Pokhilko *et al.*, 2010; see Supplementary Information). Simple models in which *CBF3* transcription was regulated by one factor consisted of three novel unconstrained parameters. For those simulating *CBF3* regulation with two clock components this number rose to 4. This increase in parameter number adds flexibility, but models of increased complexity are penalised by the AICc analysis. Models with more than three connections between the clock components and CBF3 were not considered because this radically increases the number of possible architectures. However, we make this assumption only for practical reasons and of course models with more than three connections cannot be ruled out. These models were optimised by fitting to existing time-series data describing *CBF3* expression in 12 h light/12 h dark cycles (Mockler *et al.*, 2007) using Systems Biology Software Infrastructure (SBSI, see Supplementary Information for methods). We did not consider models in which clock components modify mRNA decay kinetics because in Arabidopsis all the proteins considered in this study have been shown previously to affect transcription.

**Table 1:** Model selection by AICc, with low scores indicating the most favoured model, prefers architectures which confer regulation of CBFs by LHY, TOC1 and the Evening Complex (EC)

Rank	Model	AICc score	AICc weight	Features
1	EC↓:TOC1↓:LHY/CCA1↑	−807.29	1	Good fit to waveform and phase
2	EC↓: TOC1↓	−746.64	0	Good fit to waveform but not phase in 24L
3	EC↓:LHY/CCA1↑	−732.40	0	Good fit to waveform but not phase in 24L
4	EC↓	−700.14	0	Good fit to waveform but not phase in 24L
5	LHY/CCA1↑:TOC1↓	−316.76	0	Good fit to phase in all light dark cycles and 24L, poor fit to waveform
6	TOC1↓	−316.47	0	Good fit to phase in LD and 24L only, poor fit to waveform
7	LHY/CCA1↑	−257.29	0	Good fit to phase in LD and 24L only, poor fit to waveform
8	LHY/CCA1↑:NI↓:PRR7↓:PRR9↓	−220.30	0	Good fit to phase in LD and 24L only, poor fit to waveform
9	EC↑	−105.04	0	Good fit to neither phase or waveform
10	NI↓:PRR7↓:PRR9↓	−76.48	0	Good fit to neither phase or waveform
11	NI↓	2.80	0	Good fit to neither phase or waveform
12	PRR7↓	4.16	0	Good fit to neither phase or waveform
13	PRR9↓	10.27	0	Good fit to neither phase or waveform

AICc analysis considered in parallel matches to data in the four photoperiodic regimes outlined in Figure 2. Upward pointing arrows indicate transcriptional up-regulation of *CBF3* by preceding variable, downward pointing arrows indicate inhibition. 24L, constant light conditions; LD, long days.

### Model selection using the corrected Akaike Information Criterion

**Figure 3:**
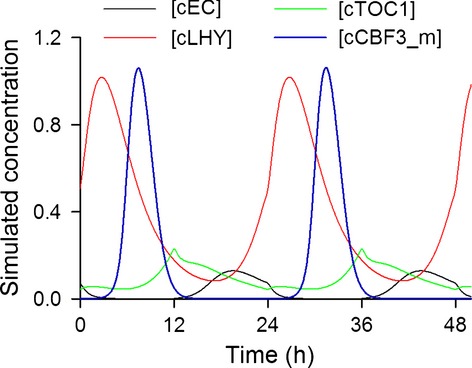
Simulated protein abundances of clock species proposed to control *CBF3* expression. Activation by LHY in the morning is first prevented by the repressive action of EC. During the afternoon loss of EC allows CBF3 expression but this declines in a feed-forward manner because the loss of LHY reduces transcription both directly and indirectly via de-repression of the repressor, TOC1. As TOC1 levels decline through the night, the role of repressor is resumed by the EC. Simulation uses 12 h light/12 h dark cycles.

AICc scores were calculated for all model variants and ranked, comparing model fit to *CBF* expression in four light regimes (see Supplementary Information; Table S1). The four highest ranking models were parameterised to closely match experimental data for *CBF3* expression in light dark cycles (Figure 2). By extending the analysis to introduce parameter uncertainty, the same four models continued to be the most probable (see Supplementary Information). These all included inhibition of expression by the Evening Complex (EC), which appeared necessary to generate the sharp peak in *CBF3* expression observed 8 h after dawn. Models without the EC inhibition could in general match phase, but not the sharpness of the waveform of expression. This included a model in which *CBF* expression was controlled by LHY alone, but although this could match phase, the broad peak of *CBF3* activation did not resemble that observed in experimental data. Models that were very unsuccessful included control by direct inhibition by PRRs, suggesting that up-regulation of *CBF3* expression in *prr5 prr7 prr9* triple mutants (Nakamichi *et al.*, 2009) is not necessarily caused by loss of direct inhibition of transcription by these genes. Importantly, models which included inhibition by the EC alone or in combination with LHY activation predicted a 4 h phase delay in *CBF3* expression in constant light that was not observed in the experimental data (Figure 2). However, the model with the highest AICc weight, inhibition by the EC and TOC1, combined with activation by LHY, was notable in its unique ability to match not only the sharp peak in *CBF3* expression but also was capable of reproducing the phase stability in light/dark cycles and constant light. This model also retains the only directly validated link from a circadian clock component to *CBF3* expression, that of CCA1 (represented in variable LHY). Thus our analysis predicted that the simple waveform of *CBF* expression requires the complex interaction of various components to reproduce oscillations seen in experimental data. In this model *CBF3* transcriptional activation by LHY is rapidly inhibited by the action of TOC1 at dusk, whereas the primary function of the EC is to repress transcription over dawn during the early period of LHY activity (Figure 3).

### Simulation of *CBF3* expression in single and multiple mutants of oscillator components

**Figure 4:**
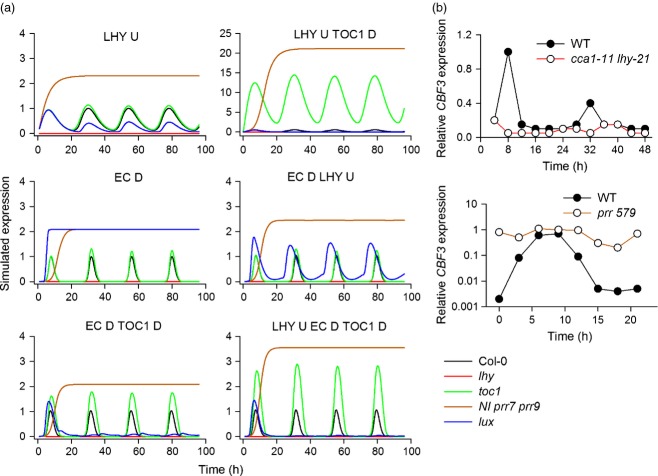
Simulation of *CBF3* mRNA expression in constant light in simulated circadian clock gene mutant backgrounds in the five most favoured models, compared with the model of the only known direct connection of the clock mechanism with CBFs, that of LHY/CCA1. Models shown are simulated in constant light. (a) Simulation of *lhy cca1* double mutants, *ni prr7 prr9* triple mutants, *toc1,* and *lux* mutants are shown in each model. D indicates transcriptional down-regulation by variable, U-, up-regulation. (b) Real data comparing *CBF3* expression in *lhy cca1* double mutants in constant light (re-drawn from Dong *et al.*, 2011), and *prr5 prr7 prr9* triple mutants in 12 h white light 12 h darkness (re-drawn from Nakamichi *et al.*, 2009) for comparison.

Successful models for the circadian control of *CBF3* expression must reproduce the known effects of clock perturbation on *CBF3* gene expression (Nakamichi *et al.*, 2009; Dong *et al.*, 2011). Therefore we tested whether model architectures qualitatively predicted experimentally determined *CBF3* expression patterns in clock mutants. The most robust predictions in the literature are those of Dong *et al*. (2011), who showed that *CBF* mRNA oscillates with only trace levels in the *lhy cca1* double mutant, and that of Nakamichi *et al*. (2009) who showed constitutive high expression of *CBF*s in *prr5 prr7 prr9* triple mutants. Our parameterisation of multiple models allowed us to understand which variants could effectively simulate the effects of known circadian clock perturbations on *CBF3* expression (Figure 4).

Interestingly, a simple model in which *CBF3* expression was controlled by LHY up-regulation was sufficient to fulfil both criteria, with expression abolished in *lhy cca1* double mutants and elevated in *ni prr7 prr9* triple mutants. In our optimised parameter set for a model including LHY up-regulation and TOC1 inhibition, the effect of PRR loss was dramatically magnified. All models including the EC inhibition of *CBF3* expression also reproduced the down-regulation of *CBF3* expression in *lhy* mutants, and the up-regulation in *prr* mutants, suggesting that these two observations alone could in principle be explained by multiple architectures involving either expression promotion by a morning-phased component or inhibition by an evening-phased component (or both). As these predictions did not prove discriminatory between different models we continued to simulate the effects of genetic perturbations with unknown effects on *CBF3* expression, specifically loss of TOC1 or LUX (essential EC component) in each of the most favoured models (Figure 4). For simulations of *toc1* mutants the behaviour of our models divided broadly into two classes. In the first class, which included LHY up-regulation alone, EC inhibition alone, and EC inhibition combined with LHY up-regulation, *CBF* expression was predicted to be similar to wild type in *toc1* mutants. The second class included models in which TOC1 directly inhibited *CBF3* expression, in combination with EC inhibition, LHY up-regulation or both. These models all predicted an increase in *CBF3* expression in *toc1* mutants of differing magnitude: LHY up-regulation in combination with TOC1 inhibition models showed a 12-fold increase in *CBF3* expression, whereas the model including EC inhibition, TOC1 inhibition and LHY activation predicts a 2–3-fold increase. Most models predicted that *LUX* loss-of-function should lead to lower *CBF3* expression, with the exception of models that relied exclusively on the EC for down-regulation, which predicted an increase. Together these results led to a hypothesis that could be tested experimentally: A modest rise in *CBF3* expression in *toc1* mutants, and a decrease in expression in *lux* mutants would imply that models including both TOC1 and EC inhibition were most accurate in simulating control of *CBF3* expression. In contrast, if *CBF3* expression is inhibited by the EC alone, we should see no change in *CBF3* expression in *toc1* mutants, and an increase in *lux* mutants. Models relying exclusively on TOC1 for inhibition of *CBF3* expression should lead to extreme overexpression of *CBF3* in *toc1* mutants.

### TOC1 and Evening Complex components are direct inhibitors of *CBF3* expression

**Figure 5:**
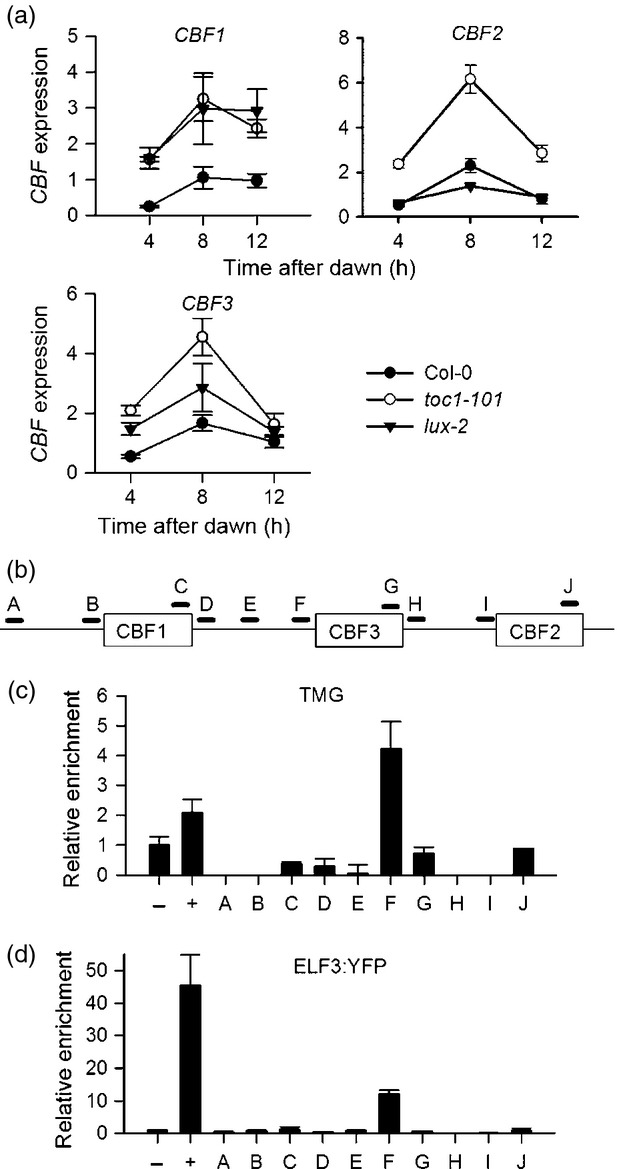
TOC1 and Evening Complex components directly regulate *CBF* transcription in Arabidopsis. (a) Real-time RT-PCR to show *CBF* expression in *toc1-101* and *lux-2* mutants compared with wild type control. Data points represent the mean and standard error of three biological replicates per genotype. (b) Cartoon to show the *CBF* locus and the location of primer pairs used for ChIP analysis. Open rectangle indicates the CBF transcribed regions horizontal lines represent regions amplified in RT-PCR. (c) ChIP using TOC1-minigene (TMG) shows that TOC1 binds a *CBF3* promoter region containing a putative t1me element (Gendron *et al.*, 2012) but not elsewhere in the *CBF* locus. For all ChIP experiments data represents the mean and standard error of three replicates per locus. −ve control ACTIN2, +ve control TOC1 binding site in the *LHY* promoter. (d) ChIP to show binding of the EC protein ELF3:YFP to a region close to the TOC1 binding site in CBF3, but not elsewhere in the *CBF* locus. −ve control ACTIN2, +ve control EC binding site in the *PRR9* promoter.

In order to probe the role of TOC1 and the EC in the control of *CBF* transcription real-time qPCR was used to examine *CBF* expression in the *toc1-101* and *lux-2* mutant backgrounds in light/dark cycles under ambient temperatures (Figure 5(a)). In our hands published *CBF1* primers also possibly mis-primed from *CBF3* (Figures S4 and S5) so expression data from these primers should be treated with caution. This analysis showed that *CBF1*, *CBF2* and *CBF3* expression were elevated approximately 2–3-fold in the *toc1-101* mutant compared with wild type. This observation supports a model architecture requiring LHY activation, with TOC1 and EC inhibition because only this model predicts an increase in *CBF3* levels in the *toc1-101* mutant consistent with the experimental data. This very same model was also favoured by AICc analysis (Table 1). The increase in *CBF* expression in *toc1-101* also effectively rules out models that do not include TOC1 as an inhibitory factor, as these models predict wild type expression levels of *CBF*s in *toc1* mutants. This prediction was general to all models without TOC1 inhibiting *CBF3* expression, rather than specific to particular parameter sets (Figure 4(a)).

We also tested the role of the EC in the control of *CBF* expression by analysis of *CBF* mRNA levels in *lux-2* mutants (Figure 5(a)). These results showed variation between the three *CBF* isoforms tested. *CBF2* showed similar expression to wild type, but *CBF1* and *CBF3* showed an increase. These results are not suggested by any of the leading models, all of which predict low *CBF3* expression in *lux* mutants, because *lux* mutants have low LHY levels (Hazen *et al.*, 2005; Pokhilko *et al.*, 2012). However, interpretation of the effect of *LUX* mutations on EC function may complicated by potential redundancy among transcription factors that can fulfil the role of LUX or cross-regulation of *CBF*s by other CBFs. Taken together, this shows that the EC has a role in the inhibition of *CBF1* and *CBF3*, expression but not *CBF2*. Our data support the predictions from our AICc analysis of our model ensemble that in addition to activation by LHY/CCA1, inhibition of *CBF* expression by both TOC1 and the EC is necessary to explain known features of the regulation of *CBF* expression by the circadian clock, but that the EC has a role in the inhibition of *CBF3*, expression but not *CBF2*. It also suggests that the P2012 model may overemphasise the role of LUX in activating LHY and CCA1 expression (see Discussion).

Previously it had been shown that TOC1 can form a complex with the PIF7 protein, and TOC1 can modify the action of PIF7, which binds the G-box element of the *CBF1* promoter (Kidokoro *et al.*, 2009). However it is unclear whether TOC1 acts directly on *CBF*s, and the *CBF3* promoter lacks the PIF7 G-box; this is not therefore a potential mechanism for the circadian control of *CBF3* expression. It has since been shown that TOC1 can function as a sequence-specific DNA-binding transcription factor *in vitro* and *in vivo* (Gendron *et al.*, 2012). Our analysis of *CBF3* promoter sequences revealed one putative copy of the recently identified TOC1 DNA-binding (t1me) element close to the transcription start site of *CBF3* which raised the possibility that TOC1 may directly bind to *CBF* promoters. Therefore chromatin immunoprecipitation (ChIP) was used to determine whether TOC1–YFP (Más *et al*., 2003) could bind at various points in the *CBF* locus, including regions across all three promoters (Figure 5(b,c)). We confirmed as a positive control that TOC1 binds to the *LHY* promoter t1me element promoter (Gendron *et al.*, 2012), and also that TOC1–YFP immunoprecipitates are enriched for the putative TOC1-binding site in the *CBF3* promoter (Figure 5(b,c)), but not in sequences downstream of *CBF3*, or in the negative control gene *ACTIN 2* (Figure 5(c)). However, we could find no further sites at which we could confirm TOC1 binding at the *CBF* locus. Therefore our statistical analysis correctly predicted that TOC1 interacts directly with *CBF3* promoter in a manner consistent with TOC1 acting as a transcriptional inhibitor, but no corresponding binding to the *CBF1* or *CBF2* loci could be detected.

We also searched the *CBF* locus for evidence of LUX binding sites (Nusinow *et al.*, 2011). No putative LUX binding sites were observed close to the transcription start site of *CBF3*, although sequences resembling published consensus LUX binding sites were observed 1.8Kb and 5Kb upstream of the *CBF3* start codon, and 1.6Kb upstream of the *CBF1* start codon. These were examined for EC binding using *ELF3:YFP* transgenic lines (Figure 5(b,d)). We could find no evidence of EC binding to the putative LUX consensus binding regions, showing that the EC does not bind this area of the *CBF1* or *CBF3* promoters. However, we could observe clear enrichment of DNA close to the TOC1 binding site in the *CBF3* promoter in ELF3:YFP immunoprecipitates (Figure 5d). These results are surprising, but raise the prospect that a modified ELF3-containing EC binds the *CBF3* promoter close to the binding site of TOC1 to impart transcriptional repression. No further sites capable of binding ELF3 were detected throughout the *CBF* locus. Taken together, these results demonstrate that multiple evening-expressed clock proteins control *CBF3* expression and that AICc analysis can be used to correctly predict connections to deterministic circadian clock models. However, they also suggest that there are other processes that provide the inhibition of *CBF1* and *CBF2* transcription at dawn as we could not find any evidence for direct binding of TOC1 or ELF3 to these promoters.

### TOC1 negatively regulates non-acclimated freezing tolerance

**Figure 6:**
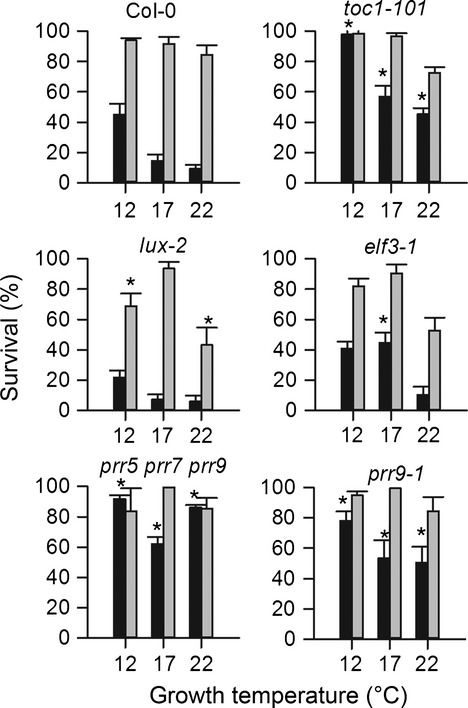
The role of TOC1 and the EC complex in freezing survival in Arabidopsis. Plants grown at either 12°C, 17°C or 22°C were frozen for 2 h at −5°C (black bars) and −3°C (grey bars) without prior cold acclimation. Data represent the mean and standard error of three independent experiments. Asterisks indicate a significant difference from wild type using Student's *t*-test (*P* < 0.01). By two-way analysis of variance (anova) both genotype (*P* = 0.0002) and growth temperature (*P* = 0.004) have highly significant effects on freezing survival.

Given that TOC1 is shown to be a negative regulator of *CBF2* and *CBF3* expression (Figure 5(a)) and that *CBF* expression induces cold acclimation and tolerance to freezing in Arabidopsis, our analysis predicts that *toc1* mutants should have an increased tolerance to freezing when grown under ambient temperatures without cold acclimation. In contrast, the potential contribution of EC components to cold tolerance is less clear. To test the freezing tolerance of evening phase clock mutants, wild type, *toc1-101, lux-2* and *elf3-1* mutants grown at three physiologically relevant temperatures were subjected to freezing stress at either −3°C or −5°C in the absence of any prior acclimation period at cool temperatures (see Experimental Procedures). At −3°C survival of all lines was high, but −5°C treatments revealed different phenotypes among the mutants. Wild type survival increased as the plants were grown at lower temperatures (Figure 6), whereas the *prr5 prr7 prr9* triple mutant showed high tolerance to freezing at all growth temperatures, as described previously (Nakamichi *et al.*, 2009). *toc1-101* mutants also showed an elevated survival rate at lower growth temperatures compared with higher temperatures, but at each growth temperature tested, *toc1-101* showed significantly increased survival compared with wild type. Thus *TOC1* is required for non-acclimated freezing tolerance in wild type plants. In contrast, *lux-2* and *elf3-1* showed freezing survival similar to wild type (Figure 6). This finding is surprising given that *lux* mutants exhibit very low *LHY* and *CCA1* expression (Hazen *et al.*, 2005), but this situation may be balanced by reduced direct inhibition of cold signalling by the EC.

### Control of *CBF3* expression by LHY, TOC1 and the Evening Complex allow observed gating of temperature signals

**Figure 7:**
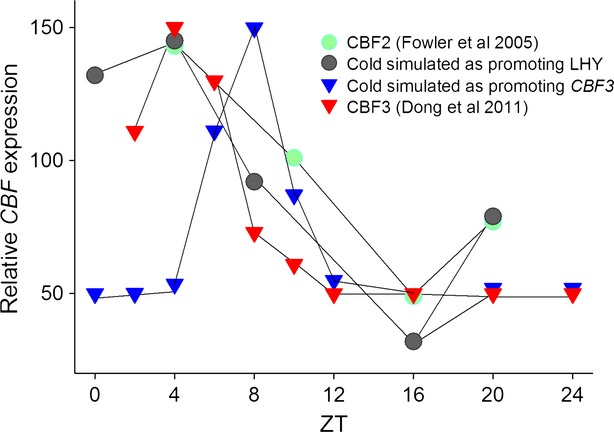
Prediction of the low-temperature gating of *CBF* expression closely matches experimental data if cold is assumed to increase the level of the LHY protein. Simulations for the model in which *CBF* expression is controlled by LHY, TOC1 and EC were scaled and plotted against data for the low-temperature induction of *CBF2* expression, gathered from Fowler *et al*. (2005; closed circles) and the cold induction of *CBF3* (Dong *et al.*, 2011). Low temperature was simulated by increasing *C*_*LHY*_ (open circles) or *C*_*CBF*__*3 *_^*m*^ (closed triangles, to simulate a cold signal arriving directly at the CBF locus) five-fold at the indicated times after subjective dawn.

Cold induction of *CBF* transcription has been proposed to be mediated by several processes. Firstly, a clock-independent signal transduction cascade involving ICE1 and/or CAMTA transcription factors has been proposed to mediate transcriptional promotion in response to cold, which is then gated by independent circadian control of *CBF* expression (Chinnusamy *et al.*, 2003; Fowler *et al.*, 2005; Doherty *et al.*, 2009). Alternatively, it has been suggested that *CBF*s receive a cold signal from the circadian clock itself, at least in part through *CCA1*, the abundance of which is affected by temperature-controlled splicing (Dong *et al.*, 2011; Seo *et al.*, 2012). To determine which of these hypotheses is supported by our model, we tested them individually. In our model in which *CBF3* expression is controlled by LHY, TOC1, and EC, this cold-regulation of CCA1 can be simulated by a pulse of LHY protein (*C*_*L*_). The effects of a five-fold increase in *C*_*L*_ on *CBF* mRNA levels were simulated every 4 h for 24 h (see Experimental Procedures). This was compared with a direct clock-independent cold induction of *CBF* transcription: as this signal is clock-independent it cannot affect the phase of *CBF* expression and therefore in this scenario cold-induced *CBF* expression follows a similar dynamic to *CBF* expression under ambient temperatures and was modelled by increasing *CBF3* levels five-fold directly. We hypothesised that the correct model for the cold induction of *CBF* expression would reproduce the observed gating of peak responsiveness of *CBF* expression to cold, a time approximating to ZT4 (Fowler *et al.*, 2005; Dong *et al.*, 2011). Fowler *et al*. (2005) used northern blot analysis to analyse *CBF* induction by cold at 4, 10, 16 or 22 h after subjective dawn; we digitised these published data to provide a quantitative analysis of the magnitude of *CBF* gene expression gating across a subjective day (Figure 7). These data were compared with the phase of cold-induced *CBF3* expression (data reproduced from Dong *et al.*, 2011), and to our model predictions. Notably, our analysis not only predicted a peak gating of *CBF3* expression in response to cold to ZT0-4 where cold induction of *CBF* occurred via LHY, but also closely replicated the magnitude of observed cold responses outside of this period (Figure 7). In contrast, circadian gating of a direct cold signal to *CBF* necessarily matches the peak phase of *CBF* expression, at ZT8, and predicts that *CBF* responsiveness to cold falls too sharply outside of the period of maximum induction. This analysis clearly shows that our simulations of temperature pulses through LHY accurately reproduce the pattern of observed cold induction whereas the pattern resulting from an external temperature signal acting additively to the circadian regulation does not. Because our model correctly predicts the phase of the gating of the transcriptional induction *CBF* expression by cold only when the temperature signal is assumed to originate within the circadian clock mechanism, we suggests that the circadian clock plays an important direct role in low-temperature signal transduction.

## Discussion

Circadian clocks play a central role in the coordination of environmental signalling pathways in plants, but given the vast numbers of clock-regulated genes simple methods are required to understand how various phased outputs are generated, and the functional significance of regulatory mechanisms. Here we extend a deterministic Arabidopsis circadian clock model and show that function connections that control *CBF3* phase and waveform can be deduced *in silico* based on the statistical analysis of alternative model architectures by AICc. Because this work uses only simple time-series datasets based on transcriptomic analyses it represents a paradigm that can be applied to biological systems in general where large public datasets of gene expression are available. Our AICc analysis correctly predicted the existence of previously unknown connections between the clock and *CBF3* expression, connections that could also be validated and shown to have physiological consequences for the plant's ability to resist cold. Our model also reproduces observed gating of *CBF* mRNA induction by cold (Fowler *et al.*, 2005), but only if the low temperature is assumed to require the clock itself for signal transduction to *CBF* expression. This finding suggests that the plant circadian clock also has a role in temperature signal transduction, as in timing biological events.

Here we have shown that in addition to the activation of *CBF* transcription by LHY and CCA1, *CBF3* transcription is also directly inhibited by at least two evening-phased components, TOC1 and ELF3, as part of the EC (Figure 5). Only models that include these three connections are able to maintain correct phase in variable photoperiods, and to qualitatively simulate the effects of mutation of clock components on *CBF3* expression. *CBF1* and *CBF2* transcription is also subject to a similar inhibition by TOC1 (Figure 5), but we could find no evidence that TOC1 binds DNA close to *CBF1* or *CBF2*. One possibility is that TOC1 binding to the neighbouring *CBF3* promoter (immediately 3′ to *CBF2*) is sufficient to confer repression, while another is that TOC1 represses *CBF1* and *CBF2* indirectly through a second factor. This factor may be via PIF7 (Kidokoro *et al.*, 2009), and it is possible that ChIP fails to detect binding via PIF7, either because the association is weak or affected by the presence of the YFP tag on the transgenic line used here. Analysis of *CBF1* expression was complicated by the very close sequence similarity between *CBF1* and *CBF3* and the possible mis-priming of published CBF1 primers from *CBF3* cDNA (Figure S4). Taken together it is clear from our data that repression by TOC1 is essential for normal *CBF* dynamics.

The EC, or some variant, also clearly binds the *CBF3* locus and inhibits *CBF3* expression (Figures5 and 6). Although this connection was predicted by our statistical analysis, it was not accurately simulated by P2012, possibly because further work is required to improve P2012, particularly to constrain the affect of EC loss on LHY and CCA1 protein levels. In our model EC loss can cause *CBF* up-regulation by loss of direct inhibition, and also down-regulation indirectly via down-regulation of LHY (Figure 1). The fact that *CBF1* and *CBF3* are up-regulated in EC mutants and *CBF2* is, if anything, slightly down-regulated shows that there is probably more than one route through which EC components can influence *CBF* expression. It is likely that, for *CBF3*, loss of direct repression dominates whereas for *CBF2* loss of LHY activation is more important, but is overestimated by the model. This highlights the differences in the regulation of the three *CBF* isoforms (Novillo *et al.*, 2004) and also suggests that there exists a yet-unknown process for providing transcriptional inhibition of *CBF2* during the night and over dawn. That ELF3 binds to a region of the *CBF3* promoter with no obvious consensus LUX binding sites suggests that the ELF3 may associate with other DNA-binding components. For instance, ELF3 has been shown to complex with other proteins, notably phytochrome, in yeast and *in vitro* (Liu *et al.*, 2001), and phytochrome has a role in the control of *CBF* expression (Franklin and Whitelam, 2007).

The *ice1* mutation also preferentially inhibits the cold induction of *CBF3*, confirming that differences in the regulation of CBF informs exist (Chinnusamy *et al.*, 2003). In addition, CBF2 has been shown to inhibit *CBF1* and *CBF3* expression after cold exposure (Novillo *et al.*, 2004) and may also contribute to inhibition of expression towards dusk, along with TOC1 activity. Our data suggest that *CBF3* expression is also the most tightly coupled to the circadian oscillator. We speculate that after duplication, pseudorandom subfunctionalisation caused by genetic drift is the most likely cause of the current specialisations of the *CBF* isoforms, in preference to invoking a particular selective advantage to the current state of regulation in Arabidopsis Col-0. In this view it is likely that the original single isoform contained most of the connections to the oscillator we observe at the *CBF3* locus, some of which have been lost at *CBF1* and *CBF2*.

Our quality control for the specificity of published *CBF* QPCR primers included expression analysis of *CBF* RNAi plants (Novillo *et al.*, 2007). From this work (Figure S4), we found that published primers detected highly overexpressed *CBF* transcripts in the RNAi lines, and that primers for *CBF1* reported elevated expression also in *CBF3* RNAi plants that had a similar expression level to that detected by the *CBF3* primers. We believe this finding indicates that the primers are most likely detecting cDNA synthesised from the overexpressed constructs, raising the suspicion that the *CBF1* primers can also prime from *CBF3* under some conditions and may not be specific. However, *CBF3* RNAi may also profoundly elevate *CBF1* expression.

Over one-third of Arabidopsis genes show a 24 h rhythm in their gene expression, suggesting control by the circadian clock (Harmer *et al.*, 2000). Our successful use of model selection and parameterisation of time-series data from public microarray data suggests that this approach may have wider utility in the extension of gene network models in other systems where large transcriptomic time-series datasets exist.

## Experimental Procedures

### Plant materials

The *lux-2* (Hazen *et al.*, 2005) and *TOC1–YFP* (Más *et al*., 2003) have been described previously and were gifts from Steve Kay. The *pELF3:ELF3:YFP* line has been described previously (Dixon *et al.*, 2011). *CBF1* and *CBF3* RNAi lines were a gift from Julio Salinas, as was the *cbf2* mutant (Novillo *et al.*, 2004, 2007). *toc1-101* was a gift from Peter Quail (Kikis *et al.*, 2005). All lines are in the wild type Columbia-0 background.

### Model construction

See the Supplementary Information for details on model construction, parameterisation and analysis.

### Gene expression and real-time PCR

Seedlings were grown for 10 days in 12 h white light/12 h dark cycles before harvest at the indicated time relative to dawn. Biological replicates were collected in triplicate and RNA extracted with an RNeasy Plant Mini Kit (Qiagen, Germantown, MD, USA) according to the manufacturer's instructions. cDNA was synthesised by Superscript Reverse Transcriptase from 3 μg total RNA using oligo-dT primers at 42°C for 1 h, and diluted 1/10 before use. Primers for the amplification of CBFs were CBF1: 5′-GGAGACAATGTTTGGGATGC-3′ and 5′-CGACTATCGAATATTAGTAACTCC-3′ (Dong *et al.*, 2011); CBF2: 5′-CGACGGATGCTCATGGTCTT-3′ and 5′-TCTTCATCCATATAAAAC GCATCTTG-3′; CBF3: 5′-AATATGGCAGAAGGGATGCT-3′ and 5′-ACTCCATAACGATACGTCGT-3′. Primers for amplification of the control gene *ACTIN2* were 5′- CGTTTCGCTTTCCTTAGTGTTA-3′ and 5′-AGCGAACGGATCTAGAGACTC-3′. PCR was performed on an ABI prism 3700 thermocycler using manufacturer's standard conditions.

### Chromatin immunoprecipitation

Chromatin immunoprecipitation was performed following the protocol in Gendrel *et al*. (2002) with modifications. In summary the following steps were altered. Seedlings were grown on MS agar plates at 22°C for 14 days with 12 hr white light/dark cycles and harvested at CT14. The chromatin was sheared to between 100 and 1000 bp in a Bioruptor UCD 200 (Diagenode) at high intensity for 10 min (cycles of 30 sec on/30 sec off) at 4°C after Lau *et al*. (2011). An aliquot of the chromatin was reserved at this point as the Input chromatin. Immunoprecipitation used equilibrated Dynabeads® Protein A (Invitrogen cat# 100-01D). The pre-cleared chromatin was transferred away from the beads and incubated with rotation over night at 4°C with a 1:1000 dilution of anti-GFP (Abcam ab290). The immunocomplexes were recovered from the beads by boiling for 10 min in the presence of 10% Chelex resin (BioRad cat# 142-1253) and the proteins removed using Proteinase K Solution (Invitrogen cat# AM2546) at 50°C. The reserved Input chromatin was also processed in parallel with Chelex and Proteinase K and then purified using QIAquick PCR purification Kit (Qiagen cat# 28104). qPCR on the ChIP and Input DNA was performed in triplicate using Brilliant III Ultra-Fast SYBR® Green QPCR Master Mix (Agilent cat# 600883) on a Mx3005P machine. Primer sequences for positive controls, the LUX binding site in the PRR9 promoter and the TOC1 binding site in the LHY promoter, have been described previously (Helfer *et al.*, 2011; Gendron *et al.*, 2012). Primer sequences can be found in Table S4.

### Analysis of freezing sensitivity

Wild type and mutant plants were grown on MS medium in 16 h light 8 h dark cycles at 22°C, 17°C or 12°C in a Sanyo MLR 350 incubator before the assay until they had two true leaves. Plants were then frozen at −5°C or −3°C for 24 h in a Sanyo MIR 154 incubator, transferred to 4°C to recover for a further 24 h, and then returned to the growth temperature for 7 days. Survival was scored as the presence of green seedlings after 7 days of growth in three biological replicates of 50 seedlings per genotype.

## Supporting Information

Table S1 Optimised new parameter values for each of the thirteen models. **Table S2.** AICc^U^ analysis results. **Table S3.** Sensitivity heatmap for the expression of *CBF3* at the indicated time after dawn. **Table S4.** Primer sequences for chromatin Immunoprecipitation.

Figure S1 Generalised method used for comparing models. **Figure S2.** Effect of *τ*^2^ **Figure S3.** Effect of *d*. **Figure S4.** Published *CBF1* Real-time PCR primers appear to prime from *CBF1* and *CBF3*. **Figure S5.** Alignment of published *CBF1*-specific primers with *CBF1* and *CBF3* cDNA sequences.
