# Supplementary material for: Model selection reveals control of cold signalling by evening-phased components of the plant circadian clock
Source: Plant J. 2013 Aug 5;76(2):247–57. doi: 10.1111/tpj.12303 (PMC4278413; doi:10.1111/tpj.12303)
Supplement: Table S1 — Optimised new parameter values for each of the thirteen models. Table S2. AICcU analysis results. Table S3. Sensitivity heatmap for the expression of CBF3 at the indicated time after dawn. Table S4. Primer sequences for chromatin Immunoprecipitation. [file tpj0076-0247-SD1.docx]

**Keily et al, Supplementary Information**

**Model Construction**

Due to the similarities in the rhythms of *CBF1-3* mRNA levels we considered constraining models to the expression of either an equivalent task. However, *CBF3* was chosen because it has higher expression levels, and because it was possible to design specific primers for RT-PCR: this was challenging for *CBF1-2* because of extremely high sequence similarity between the two genes. One equation was added to the system of ordinary differential equations that comprise the P2012 Arabidopsis clock model (Pokhilko et al, 2012) to describe the circadian regulation of *CBF3*, in the forms below, where the suffix ‘D’ to the model variant name indicates down-regulation of transcription by the protein, and the suffix ‘U’ up-regulation. Models were selected for analysis based on prior knowledge of protein function (activator or repressor or both) and prior knowledge of the affects of single and multiple loss-of-function on *CBF* gene expression. In total 13 models were analysed all consisting of P2012 plus one of the following additional statements governing possible mechanisms of control of *CBF3* expression by circadian clock components:

| TOC1D | $\frac{{dc}_{CBF3}^{m}}{dt}=n_{C1}\frac{{gC1}^{aC}}{{gC1}^{aC}+{cT}^{aC}}-m_{C1}c_{CBF3}^{m}$ |
| --- | --- |
| LHY U | $\frac{{dc}_{CBF3}^{m}}{dt}=n_{C1}\frac{{cL}^{aC}}{{gC1}^{aC}+{cL}^{aC}}-m_{C1}c_{CBF3}^{m}$ |
| LHY U:TOC1D | $\frac{{dc}_{CBF3}^{m}}{dt}=n_{C1}\frac{{gC1}^{aC}}{{gC1}^{aC}+{cT}^{aC}}.\frac{{cL}^{aC}}{{gC2}^{aC}+{cL}^{aC}}-m_{C1}c_{CBF3}^{m}$ |
| NI PRR7 PRR9D | $\frac{{dc}_{CBF3}^{m}}{dt}=n_{C1}\frac{{gC1}^{aC}}{{gC1}^{aC}+{cP7}^{aC}}.\frac{{gC2}^{aC}}{{gC2}^{aC}+{cP9}^{aC}}.\frac{{gC3}^{aC}}{{gC3}^{aC}+{cNI}^{aC}}-m_{C1}c_{CBF3}^{m}$ |
| LHY U:NI PRR7 PRR9 D | $\frac{{dc}_{CBF3}^{m}}{dt}=n_{C1}\frac{{cL}^{aC}}{{gC4}^{aC}+{cL}^{aC}}.\frac{{gC1}^{aC}}{{gC1}^{aC}+{cP7}^{aC}}.\frac{{gC2}^{aC}}{{gC2}^{aC}+{cP9}^{aC}}.$ $\frac{{gC3}^{cC}}{{gC3}^{cC}+{cNI}^{cC}}-m_{C1}c_{CBF3}^{m}$ |
| NI D | $\frac{{dc}_{CBF3}^{m}}{dt}=n_{C1}\frac{{gC1}^{aC}}{{gC1}^{aC}+{cNI}^{aC}}-m_{C1}c_{CBF3}^{m}$ |
| PRR7D | $\frac{{dc}_{CBF3}^{m}}{dt}=n_{C1}\frac{{gC1}^{aC}}{{gC1}^{aC}+{cP7}^{aC}}-m_{C1}c_{CBF3}^{m}$ |
| PRR9 D | $\frac{{dc}_{CBF3}^{m}}{dt}=n_{C1}\frac{{gC1}^{aC}}{{gC1}^{aC}+{cP9}^{aC}}-m_{C1}c_{CBF3}^{m}$ |
| EC D | $\frac{{dc}_{CBF3}^{m}}{dt}=n_{C1}\frac{{gC1}^{aC}}{{gC1}^{aC}+{cEC}^{aC}}-m_{C1}c_{CBF3}^{m}$ |
| EC U | $\frac{{dc}_{CBF3}^{m}}{dt}=n_{C1}\frac{{cL}^{aC}}{{gC1}^{aC}+EC}-m_{C1}c_{CBF3}^{m}$ |
| EC TOC1 D:LHY U | $\frac{{dc}_{CBF3}^{m}}{dt}=n_{C1}\frac{{cL}^{aC}}{{gC4}^{aC}+{cL}^{aC}}.\frac{{gC1}^{aC}}{{gC1}^{aC}+{cT}^{aC}}.\frac{{gC2}^{aC}}{{gC2}^{aC}+{cEC}^{aC}}-m_{C1}c_{CBF3}^{m}$ |
| EC D:LHY U | $\frac{{dc}_{CBF3}^{m}}{dt}=n_{C1}\frac{{gC1}^{aC}}{{gC1}^{aC}+{cEC}^{aC}}.\frac{{cL}^{aC}}{{gC2}^{aC}+{cL}^{aC}}-m_{C1}c_{CBF3}^{m}$ |
| EC D:TOC1U | $\frac{{dc}_{CBF3}^{m}}{dt}=n_{C1}\frac{{gC1}^{aC}}{{gC1}^{aC}+{cEC}^{aC}}.\frac{{gC2}^{aC}}{{gC2}^{aC}+{cT}^{aC}}-m_{C1}c_{CBF3}^{m}$ |
|  |  |
|  |  |
|  |  |

Wherein $c_{CBF3}^{m}$ is the concentration of *CBF3* mRNA; *T* the concentration of TOC1 protein, *L* the concentration of LHY protein, *P7* the concentration of PRR7 protein, *P9* the concentration of PRR9 protein, *NI* the concentration of NI protein, and *EC* the concentration of the Evening Complex. The parameters *gC1*-*gC4* are Michaelis-Menten constants, parameter *aC* Hill coefficients which were fixed to 2 (for explanation see Pokhilko et al., 2012), *n_C1_* and *m_C1_* the rate constants for mRNA synthesis and degradation respectively.

The model was optimised by comparing *CBF3* mRNA data from 12L:12D diurnal cycles (diurnal.mocklerlab.org/; Mockler et al, 2007; Figure 2) to simulated rhythms using a parallel genetic algorithm (PGA) in the model optimisation framework, SBSI Visual (http://www.sbsi.ed.ac.uk/). Parameter values for the P2012 ODEs were fixed at their published values, and values of new parameters for each model are shown in Supplementary Table 1. Maximum parameter values were set to 5: for higher values the potential for variation in variable concentrations to affect simulated *CBF3* gene expression tends to zero, and are therefore not useful for the simulation of these biological systems. *CBF3* mRNA shows peak 8 hours after dawn in this dataset, and low and invariant expression at other time-points. This background expression was not significantly different to zero and was thus the lowest expression value for *CBF3* expression in each time-series was assumed to be zero and other data-points normalised accordingly. Use of microarray rather than RT-PCR data was considered central to the aims of the project, as success opens the door to understanding the control of multiple circadian outputs for which microarray data are available, approximately 1/3 of the genome (Harmer et al, 2000). Model equations were solved and simulated using the differential equation solver CVODES (Hindmarsh et al, 2005; Serban and Hindmarsh, 2005). Parallel genetic algorithms converge to find a parameter set where the error between the simulation and data reaches a minimum (Muhlenbein et al, 1991). In our optimisation process, we set the target minimum error to be 0.01. This minimum, though, could be a local minimum in the parameter space and not the global minima. Studies have discovered that ‘sloppy’ parameters, that are not necessarily the global minima of parameter space, lead to systems that produce the correct dynamics in a number of conditions (Brown et al, 2004). Simulated annealing methods attempt to find global minima but at a greater computational cost compared to parallel genetic algorithms. Studies have shown annealing procedures are only slightly better at finding global minima compared to genetic algorithms (Laskey et al, 2003). The PGA method used here was found to take O(10^2^) iterations to converge to the fixed cost target of 0.01, compared to O(10^5^) iterations for simulated annealing.

**Model Selection**

In this study we have used model selection techniques to assess the capability of mathematical models to describe the experimental data obtained for an output of the Arabidopsis circadian clock. Model selection techniques provide objective, numerical metrics to balance competing priorities of model construction. The need for a model to describe data accurately must be weighed against the complexity of the model (Frequentist approach) that is determined by the number of parameters in the model, or the variability associated with the model (Bayesian approach) that arises from the uncertainty in the values of parameters (Akaike, 1974; Burnham and Anderson, 2004; Friel and Pettitt, 2008; Vyshemirsky and Girolami, 2008). A models accuracy and fit to the data can be improved by increasing the number of parameters. However, due to the overall increase in the uncertainty of the parameter values in a model with added parameters, the model loses its predictive power. The results of model selection analysis help to determine whether a model overfits the data as a consequence of increased complexity.

All model selection techniques are based on the calculation of likelihood probabilities, where the higher the probability, the more likely the model is able to describe the relevant experimental data (Akaike, 1974; Burnham and Anderson, 29004; Friel and Pettitt, 2008; Vyshemirsky and Girolami, 2008). Burnham & Anderson (2004) discuss a number of philosophies and pitfalls in using such techniques. Here, we describe the calculation of the corrected Akaike Information Criterion (AICc) used in this study and attempt to build on this work to offer a similar measure that takes into account the inherent uncertainty in model parameter values.

In the following sections we will use the following general notation: in j=1,…,*D* datasets there will be i=1,…,*m* datapoints, $n_{i}^{(j)}$, evaluated at certain timepoints *t_i_*; *M* denotes a model simulation from a model of *k* parameters that takes the values *M_i_ = M(t_i_)*.

***Calculation of AICc***

The Akaike Information Criterion (AIC) was first developed in the 1970’s to approximate the Kullback-Leiber (K-L) divergence (Akaike, 1974). For our case, the K-L divergence is related to the distance found between data and model simulations which we are looking to minimize. Interestingly, references point out that the K-L divergence is related to Boltzmann’s measure of entropy that is regularly used in information theory (Burnham and Anderson, 2004, Vedral, 2012). The AIC is calculated as ([1]):

| $AIC=-2\log(L_{MLE})+2k$, | (1) |
| --- | --- |

where *L_MLE_* is the maximum likelihood estimate of the likelihood function. Hurvich & Tsai (1989) showed that the AIC provided a rather poor approximation of the K-L divergence when the ratio of datapoints to parameters was large (Burnham and Anderson, 2004; Hurvich and Tsai, 2005). However, the AICc proved to be a more accurate unbiased estimator of the K-L distance and is calculated as:

| $AICc=-2\log(L_{MLE})+\frac{2q(k+1)}{q-k-2}$. | (2) |
| --- | --- |

where *q* is the total number of datapoints used in the analysis, ie. $q=Dm$. The second term of (2) always has to be positive so that the first term is correctly ‘penalised’ by the number of parameters in the model, i.e. $q>k+2$.

In (1) and (2) we have shown the formulations of AIC and AICc featuring the likelihood probability. As pointed out by Burnham & Anderson (2004), these results can be reduced in the special case where we assume the differences between a datapoint from dataset *D* and the model simulation at the same time follow a normal distribution. Hence,

| $n_{i}^{(D)}-M_{i}\sim N(0,\sigma^{2})$, | (3) |
| --- | --- |

where $\sigma^{2}=\frac{\sum_{i=1}^{m} (n_{i}^{(D)}-M_{i})^{2}}{m}$. This can be proven by using the central limit theorem with a very large number of datapoints ($m\to\infty$).

Using this assumption, the first term of the AIC and AICc reduces to

| $-2\log(L_{MLE})=m\log(\sigma^{2})$. | (4) |
| --- | --- |

Thus, the AICc used in this study is based on

| $AICc=\sum_{j=1}^{D} m_{(j)}\log(\sigma_{(j)}^{2})+\frac{2q(k+1)}{q-k-2}$, | (5) |
| --- | --- |

where *m_(j)_* is the number of datapoints in dataset *j*.

***Ensuring q > k+2 using circadian data***

As mentioned in the previous section, for the AICc analysis to accurately penalise a model by its complexity (or the number of parameters), then the number of datapoints used in the analysis has to be greater than the number of parameters add two. Since we are not comparing different models of the circadian clock, the analysis specifically requires data of our chosen clock output, *CBF3*, to determine which model is preferable. Therefore, since the circadian clock model itself features 109 parameters, we require either a very large single dataset for our output or numerous smaller data sets to ensure that *q>k+2* is satisfied. However, genes with circadian regulation should have a similar level of expression at the same time of day, or point of the limit cycle, for several days/cycles. This means we can concatenate simulations and data from one cycle such that$n^{(j)}(t_{i})=n^{(j)}(t_{i}+24(d-1))$, where *d* is the number of whole days, to ensure that the number of data points is suitably large. This means that we can take a dataset that describes 1 day to describe 3 days by repeating the dataset 3 times. For example, if we have data at *t=0,4,8,12,16,20,24* with *n(0)=n(24)* then we count the points in *t=[0,20]* three times to create three limit cycles and the *t=24* once as the final timepoint of the 3 days. Since the model simulates circadian gene expression on a limit cycle, the simulations at the respective timepoints will also be the same in day 1 as day 3. As we have concatenated the data for both AICc and AICc^U^, the later discussions comparing results from the two methods are independent of the data repetition used here to ensure *q>k+2*. Figure S1 shows an example of a model and dataset in 12L:12D cycles. Hence, we can adapt the AICc to ensure that the penalty term is always positive:

| $AICc=d\sum_{j=1}^{D} (m_{(j)}-1)\log(\sigma_{(j)}^{2}\left\vert_{t\neq24}\right.)+\sum_{j=1}^{D} \log(\sigma_{(j)}^{2}\left\vert_{t=24}\right.)+\frac{2(d\sum_{j=1}^{D} (m_{(j)}-1)+D)(k+1)}{d\sum_{j=1}^{D} (m_{(j)}-1)+D-k-2}$ | (6) |
| --- | --- |

where *d* is the number of days required to ensure that $d\sum_{j=1}^{D} (m_{(j)}-1)+D>k+2$ and $\log(\sigma_{(j)}^{2}\left|_{t=i}\right.)$ is the value of $\log(\sigma_{(j)}^{2})$ evaluated for the *t=i* timepoint. The third term of this equation shall be referred to as the ‘penalty’ term

| $Z=\frac{2(d\sum_{j=1}^{D} (m_{(j)}-1)+D)(k+1)}{d\sum_{j=1}^{D} (m_{(j)}-1)+D-k-2}$. |  |
| --- | --- |

***Akaike Weights***

Whereas AICc scores can generally take on a wide range of values, Akaike Weights use the AICc scores to provide a probability measure for a model variant from the set of models (Burnham and Anderson, 2004). Thus, the higher the probability for a given model, *M*, the more likely it is that *M* fits the data without overfitting the data. This provides us with our measure of deciding which model variant from our set is favoured. They are calculated as

| $p(M)=\frac{\exp\{-\Delta_{s}/2\}}{\sum_{r} \exp\{-\Delta_{r}/2\}}$ | (7) |
| --- | --- |

where $\Delta_{s}=AICc_{s}-AICc_{\min}$ is the relative AICc score and *AICc*_min_ is the minimum value of AICc from the set of *r* models that corresponds to the ‘best’ model ([1],[2],[5]). Values of $\Delta_{s}>10$ imply that a model does not have any statistical support. If a model has a value of $\Delta_{s}<10$ then the model has statistical support, with the significance increasing as $\Delta_{s}\to0$ (Burnham and Anderson, 2004). These values thus show how much more likely one model is favoured compared to another.

***Model Uncertainty in AICc (AICc^U^)***

The AICc is generally seen as a Frequentist measure by statisticians as it supposes that the value of the parameters, *k_l_*, are fixed. In comparison, Bayesian statistical inference includes the uncertainty associated with parameter values of a model. The reason for this is that the important properties of a specific model are expected to be robust such that they are maintained over a range of parameter values (for example see Song et al, 2012; Pokhilko et al, 2012). Hence, the ‘true’ parameter value lies somewhere within this range but we are uncertain of the exact value (see Supplementary Figure 1). To characterise this uncertainty, Bayesian inference looks to calculate the posterior distribution such that

***Posterior Prior* x *Likelihood***

where the prior distribution is the probability distribution of the model/parameter variability and the likelihood is the same as that discussed previously.

As outlined in Burnham & Anderson (2004), Bayesian approaches to AIC measures (for example, the Bayesian/Schwarz Information Criterion; B/SIC) assume that the ‘true’ model is one of the variants found in our set of possible models, which we cannot assume here. However, since the AICc and BIC both have a close relationship to likelihood probabilities, we rationalised that if we could approximate the uncertainty in parameter values through a prior probability, then we could include a further term to the calculation of AICc scores. In doing this, the new AICc scores (termed AICc^U^ below) would not only take into account the number of parameters used in the model, but also the uncertainty in their parameter values.

Since the calculation of prior distributions can become quite complicated (we evaluated methods such as Thermodynamic Integration found in Friel and Pettitt (2008) and Vyshemirsky, and Girolami (2008)), we made the assumption that the simplest model to describe the data was a sine curve due to the circadian rhythm of the output gene expression. In our example for this study, the circadian regulation of *CBF3* mRNA was analysed. As our models were optimised to data from 12(hours) L:12(hours) D cycles, we ensured that the peak of the sinewave matched the peak of the data from the same conditions (at ZT8; see Figure S1). We then assumed, as with the difference between the datapoints and model simulations earlier, that the differences between the models in 12L:12D cycles and our prior model followed a normal distribution. Hence, our prior distribution is defined as

| $M\sim N(\mu,\tau^{2})$ | (8) |
| --- | --- |

where $\mu$ is the value of the sinewave at *t_i_* and $\tau^{2}$is the variance calculated in the same manner as $\sigma^{2}$ (see later discussion). Therefore, the full AICc^U^ is

| $AICc^{U}=AICc+\log(\tau^{2})+\frac{\sum_{i=1}^{m} (M_{i}-\mu)^{2}}{\tau^{2}}$ | (9) |
| --- | --- |

where AICc is calculated from (6). From (9), when $\tau^{2}\to\infty$, $\Delta_{s}^{AICc}=\Delta_{s}^{AICcU}$ since the last term will tend to zero and the logarithmic term will become a constant that will disappear when $\Delta_{s}$ values are calculated.

***Results of Analysis***

The results for the AICc analysis are included in the main text (see Table 1). Here we will summarise and discuss the results of the AICc and AICc^U^ analysis, paying particular attention to the values of the number of data cycles, *d*, and the variance in model simulations due to perturbed parameter values, $\tau^{2}$, used.

*Does the prior distribution change conclusions drawn from AICc?*

Supplementary Table 2 shows the AICc^U^ scores using a value of *d* = 4 and allowing $\tau^{2}$ to be calculated in the same way as $\sigma^{2}$ for each model. Comparing these results to Table 1 shows that the same model (EC TOC1 D: LHY U) is favoured by using the AICc^U^ analysis. To test whether this result was a consequence of maintaining the use of the *Z* penalty term in the AICc^U^ analysis, we carried out the analysis again without penalising models for complexity. From Supplementary Table 2, we observed that removing *Z* did not affect which model was selected as best from the set, leading to stronger support for EC TOC1 D: LHY U as seen by the Δ_s_ values. This suggests that the same result can be achieved by penalising a model for the number of parameters in the system or by calculating a prior distribution for parameter uncertainty. However, due to the increased support of EC TOC1 D: LHY U this result also suggests that prior distributions may not penalise model complexity stringently enough, allowing one of the most complex models to dominate the result. Due to these observations made from Supplementary table 2, we based our results on the AICc analysis (Table 1, main text) that did not require prior information on model structure and had a stronger penalty for model complexity.

*Does the value of* $\tau^{2}$*affect the results?*

Over a range of $\tau^{2}$ values, the most probable model from the AICc^U^ analysis did not change and EC TOC1 D: LHY U was always selected as the most probable model. However, the likelihood of other models relative to this favoured model did change over the range of $\tau^{2}$. Supplementary Figure 2 shows how the Δ_s_ of EC TOC1 D and EC D: LHY U - the two ‘closest’ models to EC TOC1 D: LHY U - change with $\tau^{2}$ for *d* = 4. From the figure, we observed that the conclusions drawn from the analysis were unaffected by the value of $\tau^{2}$ such that Δ_s_ > 0 for all $\tau^{2}$ implying that EC TOC1 D: LHY U would always be selected as the best model from the set. However, this may be a special case, and the value of $\tau^{2}$ may play a larger role in determining which model was most suitable if the analysis had been run on a set of models where the differences in accuracy to data were smaller. In particular, values of Δ_s_ seem to change more dramatically as $\tau^{2}\to0$.

*How does the value of d affect results?*

As the total number of datapoints that was being used in the analysis was $\sum_{j=1}^{D} (m_{(j)}-1)+D=98$ and the largest model contained *k* = 130 parameters the minimum value of *d* that could be used was *d* = 2 to ensure that $d\sum_{j=1}^{D} (m_{(j)}-1)+D>k+2$. From Supplementary Figure 3a, we observed that when *d* < 3, EC D is selected as the suitable model from the set using AICc. This model contains *CBF3* regulation solely through repression from the evening complex (EC) of the circadian clock. When *d* ≥ 3, the analysis concludes that EC TOC1 D: LHY U is the most appropriate system for *CBF3* regulation. The increase in *d* leads to an increase in the number of data points considered in the analysis (see above), which in turn has been shown to improve the accuracy of AICc scores in comparison to the true Kullback-Leiber divergence (Hurvich and Tsai, 1989). The results quoted in Table 1 and Supplementary Table 2 are for the case where *d = 4*.

The reason for the change in results with the change in *d* can be observed from Supplementary Figure 3 where the difference between *Z*-values of EC TOC1 D: LHY U (*k*=128) and EC D (*k*=124) has been calculated. This shows that EC TOC1 D: LHY U is penalised more than EC D regardless of *d*. However, as *d* increases, the increased penalty for EC TOC1 D: LHY U decreases relative to the penalty for EC D, i.e. the difference between the two terms decreases. This means that the difference in the AICc values for EC TOC1 D: LHY U and EC D would become more dependent on the accuracy of the simulations compared to the data. Since EC TOC1 D: LHY U is more accurate than EC D ($\sum_{j=1}^{D} m_{(j)}\log(\sigma_{(j)}^{2})+D\approx-320.4<-290.7$, where the lower value implies accuracy), this difference would be amplified as *d* increases, overcoming the difference in penalty terms.

**Model analysis**

To simulate circadian clock null mutants the corresponding protein levels were fixed at zero. Gating by low temperature was simulated by assuming a 5-fold increase in either LHY nuclear protein or *CBF* mRNA levels as indicated. Simulations were compared to data presented in Figure 2A from Fowler et al, (2005). Northern blot image was imported in Photoshop (Adobe systems) and maximum *CBF* expression after cold at each time point calculated using the histogram function, and are presented in arbitrary units. Simulation outputs were scaled such that the maximum values of *CBF* expression were equivalent to the maximum value in the observed data. Although the observed data was obtained by hybridisation to a *CBF2* probe, experiments elsewhere in Fowler et al, (2005) show that *CBFs1-3* behave similarly. In order to check that the performance of our model of *CBF3* regulation by LHY, TOC1 and EC is robust to parameter variation we performed sensitivity analysis using COPASI, analysing the fold change in *CBF3* expression at 4 hour intervals across one light dark cycle with a delta factor of 0.001 and a delta minimum of 1e-12. (Supplementary Table 3). This showed that parameters that govern the relationship of LHY, TOC1 and EC with CBF3 mRNA levels were among the least sensitive in the model, the most sensitive being the degradation rate of *CBF3* mRNA, and parameters governing the light sensitivity of the clock mechanism. That an appropriate *CBF3* degradation rate is essential for the fast fall in *CBF* levels after transcriptional inhibition is not surpising, and the model was only sensitive to *CBF3* degradation rate in the hours following the period of peak expression. We therefore conclude that the core properties of our model are not manifest with a small unique parameter range, and that the model is robust to local variation of new parameter values, with the exception of *CBF3* degradation rates, which must be fast.

**Supplementary References**

**Akaike H.** (1974) A new look at the statistical model identification. *IEEE Transactions on Automatic Control* **19**: 716-723

**Burnham KP, Anderson DR.** (2004) Multimodel inference: Understanding AIC and BIC in model selection. *Sociological Methods Research* **33**: 261-304.

**Dong MA, Farre I. and Thomashow MF.** (2011) CIRCADIAN CLOCK-ASSOCIATED 1 and LATE ELONGATED HYPOCOTYL regulate expression of the C-REPEAT BINDING FACTOR (CBF) pathway in Arabidopsis. *PNAS* **108**: 7241-7246

**Fowler SG, Cook D, Thomashow MF.** (2005) Low temperature induction of Arabidopsis CBF1, 2, and 3 is gated by the circadian clock. *Plant Physiol* **137**: 961-968

**Friel N, Pettitt AN.** (2008) Marginal likelihood estimation via power posteriors. *Journal of the Royal Statistical Society B* **70**: 589-607

**Harmer SL, Hogenesch JB, Straume M, Chang HS, Han B, Zhu T, Wang X, Kreps JA, Kay SA.** (2000) Orchestrated transcription of key pathways in Arabidopsis by the circadian clock. *Science* **290**: 2110-2113

**Hindmarsh AC, Brown PN, Grant KE, Lee SL, Serban R.** (2005) SUNDIALS: Suite of nonlinear and differential/algebraic equation solvers. *ACM Transactions on Mathematical Software* **31**: 363-396

**Hurvich CM, Tsai CL.** (1989) Regression and time series model selection in small samples. *Biometrika* **76**: 297-307

**Laskey KB, Myers JW.** (2003) Population markov chain monte carlo. *Machine Learning* **50**: 175-196

**Mockler TC, Michael TP, Priest HD, Shen R, Sullivan CM.** (2007) The DIURNAL project: Diurnal and circadian expression profiling, model-based pattern matching and promoter analysis. *Cold Spring Harbor Symposium Quantitative Biology* **72**: 353-363

**Muhlenbein H, Schomisch M, Born J.** (1991) The parallel genetic algorithm as function optimizer. *Parallel Computing* **17**: 619-632

**Novillo F, Medina J, Salinas J.** (2007) Arabidopsis CBF1 and CBF3 have a different function than CBF2 in cold acclimation and define different gene classes in the CBF regulon. *Proc. Natl. Acad. Sci. USA*, 104, 21002-21007.

**Pokhilko A *et al.*** (2012) The clock gene circuit in *Arabidopsis* includes a repressilator with additional feedback loops. *Molecular Systems Biology* **8**: 574

**Serban R, Hindmarsh AC**. (2005) CVODES: the sensitivity-enabled ODE solver in SUNDIALS. *Proceedings of IDETC/CIE*

**Song YH *et al.*** (2012) FKF1 conveys timing information for CONSTANS stabilization in photoperiodic flowering. *Science* **336**: 1045-1049.

**Vedral V.** (2012) *Decoding reality: The universe as quantum information*. Oxford University Press.

**Vyshemirsky V, Girolami MA.** (2008) Bayesian ranking of biochemical system models. Bioinformatics 24: 833-839.

**Supplementary Tables**

| **Model** | **Parameter** | **Value** | **Model** | **Parameter** | **Value** |
| --- | --- | --- | --- | --- | --- |
| TOC1D | *mC1* | 0.2746 | PRR9 D | *mC1* | 5.0000 |
|  | *nC1* | 5.0000 |  | *nC1* | 0.9475 |
|  | *gC1* | 0.0081 |  | *gC1* | 5.0000 |
|  | *aC* | 2.0000 |  | *aC* | 2.0000 |
| LHY U | *mC1* | 0.2176 | EC D | *mC1* | 2.3963 |
|  | *nC1* | 0.2419 |  | *nC1* | 5.0000 |
|  | *gC1* | 0.7667 |  | *gC1* | 0.0002 |
|  | *aC* | 2.0000 |  | *aC* | 2.0000 |
| LHY U TOC1 D | *mC1* | 0.2350 | EC U | *mC1* | 0.0250 |
|  | *nC1* | 4.9751 |  | *nC1* | 4.7871 |
|  | *gC1* | 0.0103 |  | *gC1* | 1.9305 |
|  | *gC2* | 0.4754 |  | *aC* | 2.0000 |
|  | *aC* | 2.0000 |  |  |  |
| PRR7 PRR9 NI D | *mC1* | 0.0583 | LHY U EC TOC1 D | *mC1* | 1.4070 |
|  | *nC1* | 0.5224 |  | *nC1* | 4.9967 |
|  | *gC1* | 5.0000 |  | *gC1* | 0.0434 |
|  | *gC2* | 5.0000 |  | *gC2* | 0.0005 |
|  | *gC3* | 0.0270 |  | *gC4* | 0.1344 |
|  | *aC* | 2.0000 |  | *aC* | 2.0000 |
| LHY U NI PRR7 PRR9 D | *mC1* | 0.2059 | LHY U EC D | *mC1* | 2.0390 |
|  | *nC1* | 0.2608 |  | *nC1* | 5.0000 |
|  | *gC1* | 5.0000 |  | *gC1* | 0.0003 |
|  | *gC2* | 5.0000 |  | *gC2* | 0.4020 |
|  | *gC3* | 5.0000 |  | *aC* | 2.0000 |
|  | *gC4* | 0.8186 |  |  |  |
|  | *aC* | 2.0000 |  |  |  |
| NI D | *mC1* | 0.3682 | EC TOC1 D | *mC1* | 1.9056 |
|  | *nC1* | 0.0711 |  | *nC1* | 3.9652 |
|  | *gC1* | 5.0000 |  | *gC1* | 0.0004 |
|  | *aC* | 2.0000 |  | *gC2* | 0.0723 |
| PRR7 D | *mC1* | 0.2683 |  | *aC* | 2.0000 |
|  | *nC1* | 0.0511 |  |  |  |
|  | *gC1* | 5.0000 |  |  |  |
|  | *aC* | 2.0000 |  |  |  |

**Supplementary Table 1.** Optimised new parameter values for each of the thirteen models.

| **Model** | **With *Z*** | | | **Without *Z*** | | |
| --- | --- | --- | --- | --- | --- | --- |
|  | AICc^U^ | $\Delta_{s}$ | % | AICc^U^ | $\Delta_{s}$ | % |
| TOC1D | -301.9 | 432.3 | 0% | -692.5 | 452.1 | 0% |
| LHY D | -234.1 | 500.2 | 0% | -624.7 | 520.0 | 0% |
| LHY U: TOC1↓ | -284.8 | 449.4 | 0% | -685.2 | 459.4 | 0% |
| NI PRR7 PRR9 D | -102.6 | 631.4 | 0% | -503.0 | 641.4 | 0% |
| LHY U: NI PRR7 PRR9 D | -199.8 | 534.6 | 0% | -620.4 | 524.4 | 0% |
| NI D | -26.4 | 707.0 | 0% | -417.0 | 726.9 | 0% |
| PRR7 D | -25.0 | 708.4 | 0% | -415.6 | 728.2 | 0% |
| PRR9 D | -18.1 | 715.2 | 0% | -408.8 | 735.1 | 0% |
| EC D | -634.7 | 99.6 | 0% | -1025.3 | 119.5 | 0% |
| EC U | -141.7 | 591.8 | 0% | -532.3 | 611.7 | 0% |
| EC TOC1 D: LHY U | -734.4 | 0.0 | 100% | -1144.8 | 0 | 100% |
| EC D: LHY U | -665.9 | 68.5 | 0% | -1066.3 | 78.5 | 0% |
| EC TOC1 D | -678.6 | 55.8 | 0% | -1079.0 | 65.8 | 0% |

**Supplementary Table 2: AICc^U^ analysis results.** Analysis was run with and without the penalising of the number of model parameters (2^nd^ term in (9)). *d* = 4.

| **Process** | **parameter** | **fold-change in CBF3 expression** | | | | | | |
| --- | --- | --- | --- | --- | --- | --- | --- | --- |
|  |  | **0** | **4** | **8** | **12** | **16** | **20** | **24** |
| degradation rate of CBF3 mRNA | *mC1* | -0.6753 | -0.4788 | -0.9705 | -3.0172 | -7.5791 | -2.9703 | -0.6751 |
| translation of COP1 | *N5* | 1.2679 | 5.3098 | -0.4674 | -0.4786 | -0.3550 | 0.9452 | 1.4847 |
| light induced degradation of LHY mRNA | *m1* | 1.9480 | 4.5889 | 0.1102 | -3.7692 | -4.2381 | -1.0835 | 1.0093 |
| affect of light on COP1 conformation | *m37* | 0.0888 | 4.0990 | 0.0404 | -0.0086 | 0.01102 | 0.03799 | 0.09762 |
| light induced affect of GI on EC | *m32* | 0.8533 | 3.7632 | 0.01307 | 0.4296 | 0.4341 | 0.3416 | 0.9600 |
| inhibition of toc1 by LHY | *g5* | -1.2378 | -1.7304 | -2.3497 | -3.4919 | -3.3917 | -1.8436 | -1.0352 |
| light induced affect of GI on EC | *g7* | -0.0186 | -3.4220 | -0.1422 | 0.07550 | 0.0610 | -0.0119 | -0.0167 |
| degradation rate of LHYmod | *m4* | 1.0758 | 1.8731 | 1.8610 | 3.1939 | 3.2043 | 0.8864 | -0.0038 |
| light-independent degradation of LHY | *m3* | 1.1209 | 3.0383 | 0.5239 | -2.5014 | -3.1836 | -2.1718 | 0.0500 |
| light-induced degradation of LHY protein | *g3* | 0.4061 | 0.3210 | 0.9196 | 2.8403 | 3.1639 | 1.5340 | 0.0974 |
| degradation of LHY protein | *p3* | -0.1124 | 0.1388 | -0.8612 | -2.813 | -3.0431 | -1.2100 | 0.0763 |
| LHY inhibition by PRRs | *g1* | 1.1323 | 1.9246 | 1.5942 | 2.8389 | 2.9078 | 1.5544 | 0.40368 |
| binding of ELF3 to GI | *p17* | -1.7329 | -2.6658 | 0.0761 | 1.4649 | 1.7247 | -0.9330 | -1.6330 |
| inhibition of GI by LHY | *g15* | -0.7633 | 2.6284 | 0.8540 | 1.5771 | 1.6236 | -0.2328 | -0.7312 |
| nucleocytoplasmic transport of GI protein | *p28* | 0.65076 | 2.4337 | 0.2818 | -0.1579 | -0.3288 | 0.1850 | 0.5927 |
| rate constant for TOC1 transcription | *n2* | -1.3576 | -1.6208 | -1.6313 | -2.4036 | -2.4317 | -1.8960 | -1.1214 |
| rate constant for TOC1 translation | *p4* | -1.3523 | -1.6100 | -1.6264 | -2.3963 | -2.4244 | -1.8961 | -1.1239 |
| degradation of NI mRNA | *m16* | 1.1784 | 2.3782 | 1.2444 | 1.8116 | 1.8224 | 1.1574 | 0.5332 |
| light induced LHY transcription | *q1* | -0.9019 | -2.2764 | 0.2689 | 1.8124 | 1.9007 | 0.1975 | -0.5603 |
| inhibition of ELF4 translation by LHY | *g6* | 1.1336 | 0.5166 | -0.4290 | -2.0977 | -2.2378 | 0.3279 | 0.9528 |
| degradation of ELF4 mRNA | *m34* | 0.8702 | 2.1391 | -0.4776 | 0.2212 | 0.4955 | 0.8042 | 1.1906 |
| degradation of GI mRNA | *m18* | 2.1206 | -1.0163 | -0.0652 | -1.4907 | -1.9199 | 0.8764 | 1.9680 |
| degradation of light sensitive protein P | *m11* | 1.0535 | 0.4039 | -0.1365 | -1.9519 | -2.1104 | -0.2475 | 0.6498 |
| post-translational modification of COP1 | *m31* | -0.0179 | -2.1085 | -0.1997 | -0.2366 | -0.2280 | -0.0082 | -0.0307 |
| rate constant for GI transcription | *n12* | -2.0156 | -0.6084 | 0.1940 | 1.6947 | 2.0715 | -0.8569 | -1.8794 |
| rate constant for GI translation | *p11* | -1.995 | 1.4163 | 0.1676 | 1.6329 | 2.0267 | -0.8339 | -1.8603 |
| light induced translation of GI | *q2* | 0.01949 | 2.0248 | -0.0260 | -0.0609 | -0.0434 | 0.0219 | 0.0186 |
| CBF3 inhibition by EC | *gC2* | 2.0010 | 1.9368 | 0.4040 | 1.1286 | 1.2957 | 1.8726 | 2.0009 |
| CBF3 inhibition by TOC1 | *gC1* | 1.0840 | 1.1167 | 1.2208 | 1.7306 | 1.7594 | 1.6650 | 1.0844 |
| CBF3 activation by LHY | *gC4* | -0.1771 | -0.0379 | -0.1286 | -0.4256 | -0.5555 | -1.0514 | -0.1778 |

**Supplementary Table 3.** Sensitivity heatmap fort the expression of *CBF3* at the indicated time after dawn. Values indicate the fold-change in *CBF3* mRNA value with respect 4 hour intervals where dawn is 0 and 24 hours. The 20 most sensitive parameters are shown, in addition to the parameters gC2, gC1 and gC4 which govern regulation of *CBF3* by *EC*, *TOC1* and *LHY*. This analysis shows that the model is robust to variation in new parameter values, with the exception of the *CBF3* mRNA degradation rate constant. Fast degradation is necessary to produce the sharp waveform observed. Increases in expression are shown in blue with –ve values, decreases in orange with +ve values.

| **Position in Figure 5B** | **Locus** | **Sequence** |
| --- | --- | --- |
| A | CBF1 5’ | AAAAGTCTTGCAACTTAACACTCTCA |
| A | CBF1 5’ | TGTTCGTGGCCACATATCAT |
| B | CBF1 5’ | GACGGGTGACAATTAATGACAAT |
| B | CBF1 5’ | ATATTGGCCGGAGGAGAGAT |
| C | CBF1 ORF | GGAGACAATGTTTGGGATGC |
| C | CBF1 ORF | CGACTATCGAATATTAGTAACTCC |
| D | CBF3 5’ | TTTAGCAACAGAAAGCCACAAA |
| D | CBF3 5’ | AGTGAACTGGGCTGAATTTTT |
| E | CBF3 5’ | GTTTAAACACAGCAGGAAGTAAATTAT |
| E | CBF3 5’ | TCGGAAGTCAAAATAAAAAGCA |
| F | CBF3 5’ | TGAATAACGGTTACCCTACACC |
| F | CBF3 5’ | AGTTTTATAAACTCTTTGCGCGTATGAA |
| G | CBF3 ORF | AATATGGCAGAAGGGATGCT |
| G | CBF3 ORF | ACTCCATAACGATACGTCGT |
| H | CBF3 3’ | GATGACGACGTATCGTTATGGA |
| H | CBF3 3’ | GGTTTTGCTGAATCGGTTGT |
| I | CBF25’ | TGCACGATATGTGAATGGAGA |
| I | CBF2 5’ | TCAAGGCTGTCAATCACTGAG |
| J | CBF2 ORF | CGACGGATGCTCATGGTCTT |
| J | CBF2 ORF | TCTTCATCCATATAAAACGCATCTTG |
| -VE control | ACT2 | CGTTTCGCTTTCCTTAGTGTTA |
| -VE control | ACT2 | AGCGAACGGATCTAGAGACTC |

**Supplementary Table 4.** Primer sequences for chromatin Immunoprecipitation. Positions for each primer pair are shown in Figure 5B.

**Supplementary Figures**


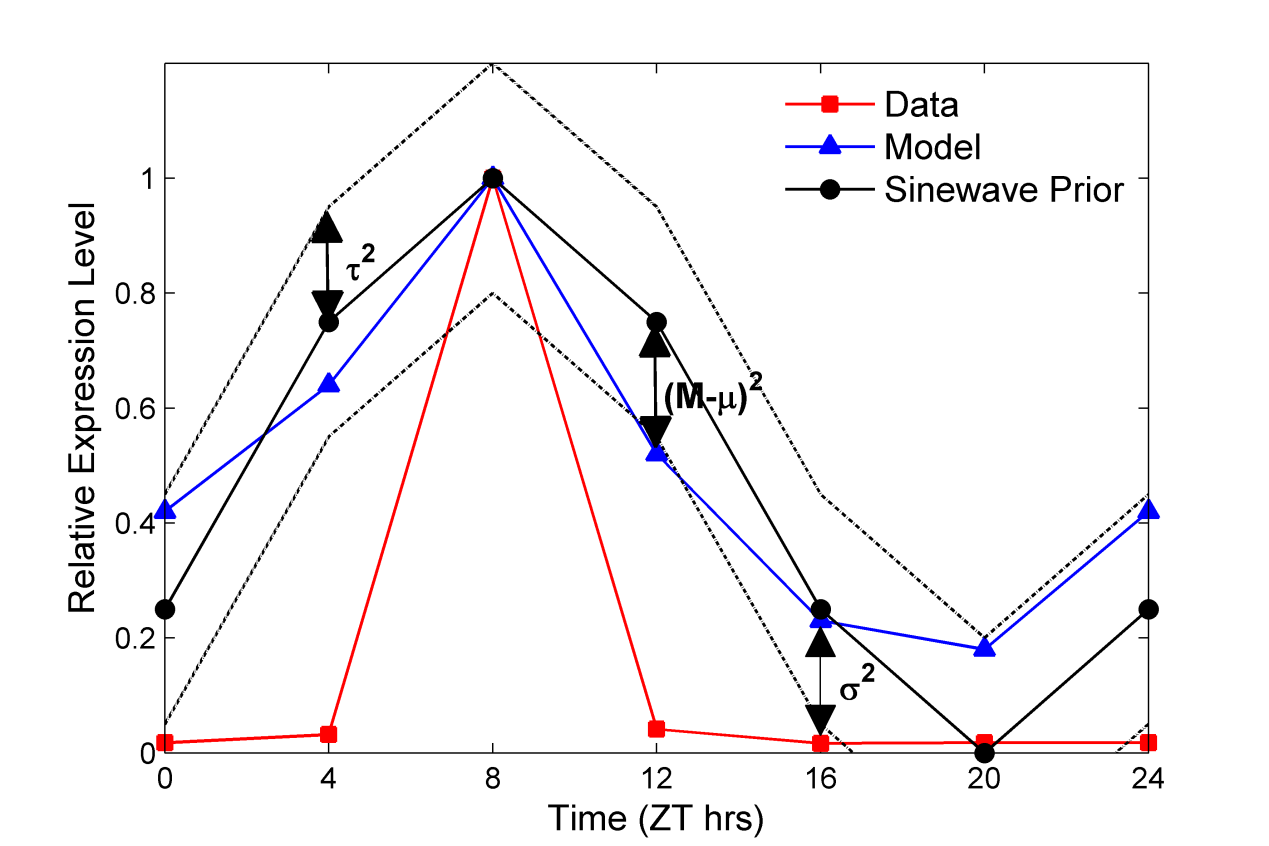


**Figure S1: Generalised method used for comparing models.**  Models were fitted to data (red dashes, squares) with the resulting example simulation (blue, triangles) in 12 hrs light (12L):12 hrs dark (12D) cycles. $\sigma^{2}$ was calculated from the difference between the model simulations and datapoints at the specific times of the day. A prior model was constructed using a sinewave (black line). The difference between the models and prior model was calculated at the same timepoints as the models were compared to the data, $\left(M-\mu\right)^{2}$. The region around the prior model sinewave (grey dashes) represents the space in which a model may lie given a perturbation to the prior model parameters. This space is characterised by the uncertainty in parameter values, $\tau^{2}$.


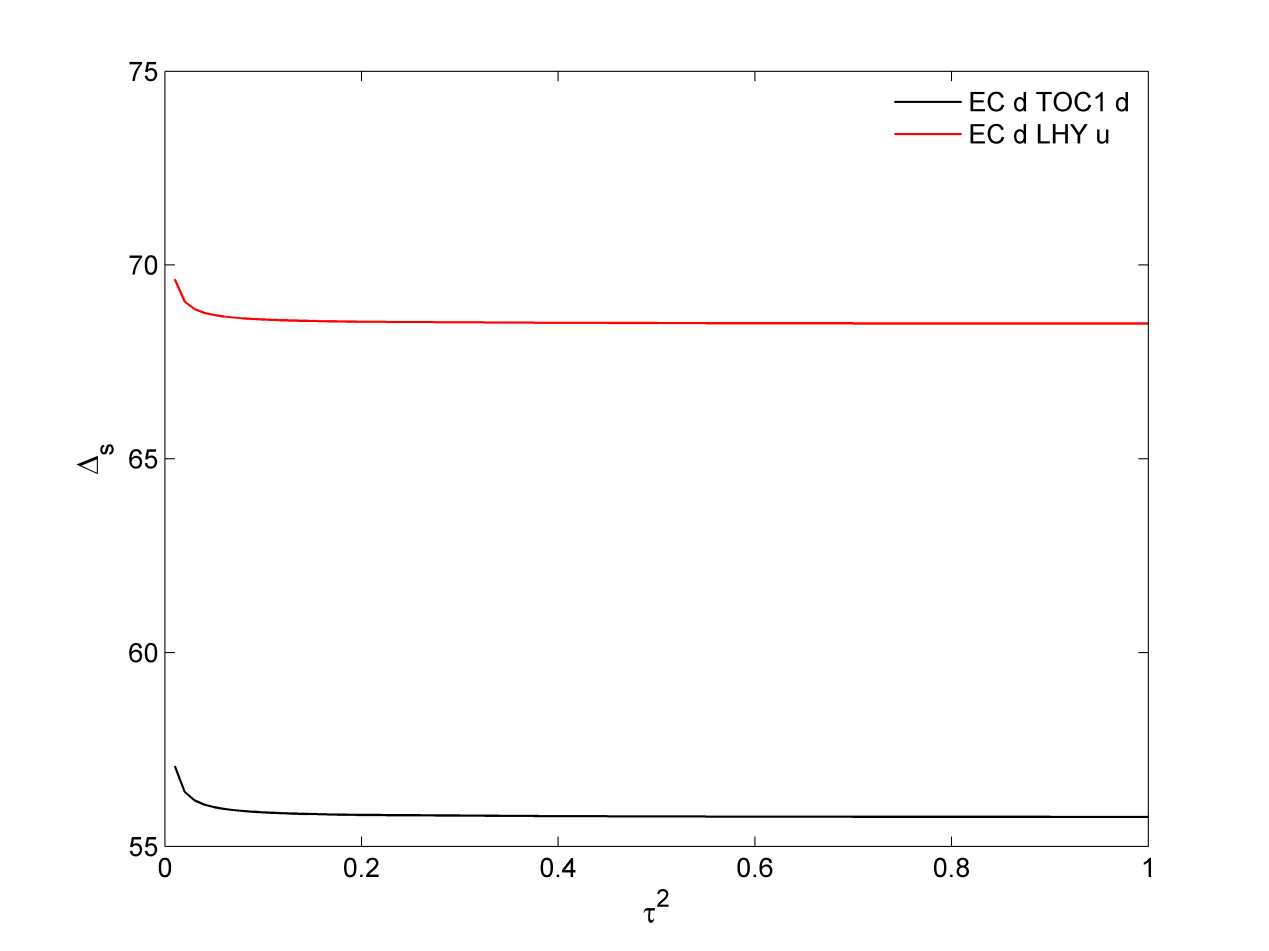


**Figure S2: Effect of $\tau^{2}$.** The AICc^U^ analysis was carried out over a range of $\tau^{2}$ values (*d* = 4). Δ_s_ values for EC TOC1 D (black line) and EC D: LHY/CCA1 U (red line) show that there would be no change in which model was favoured over the range of $\tau^{2}$ values. If the lines crossed, then that would suggest that conclusions drawn from the analysis would change at a specific value of $\tau^{2}$.


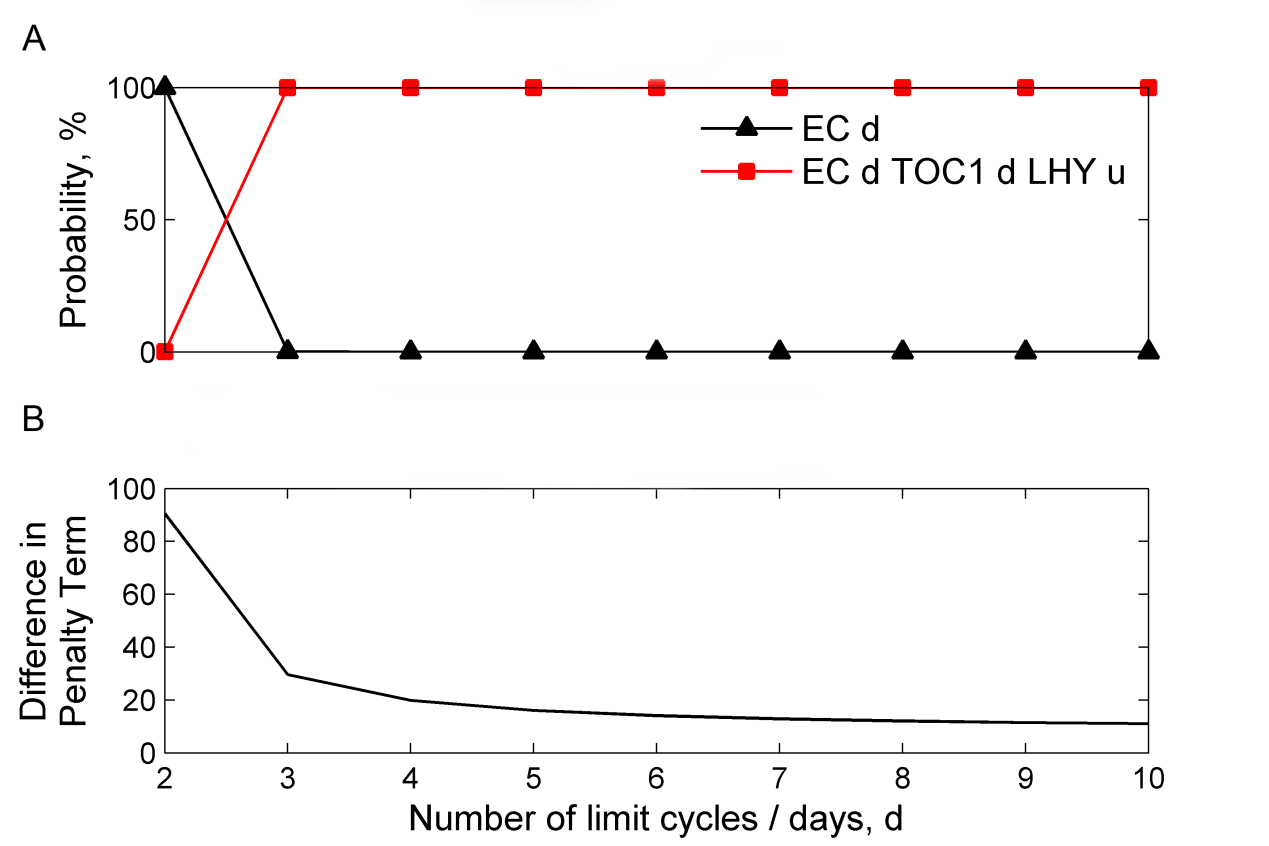


**Figure S3: Effect of *d*. (a)** The AICc analysis was carried out over a range of *d* ≥ 2. When *d* < 3, EC D was more favoured than EC TOC1 D: LHY U. When *d* ≥ 3, EC TOC1 D: LHY U was the most probable model. d = repression/ downregulation; u = activation/ upregulation. **(b)** The difference between the penalty terms of EC TOC1 D: LHY U and EC D decreases as *d* is increased. This was calculated by taking the value of *Z* from (6) and subtracting (EC TOC1 D: LHY U – EC D).

**
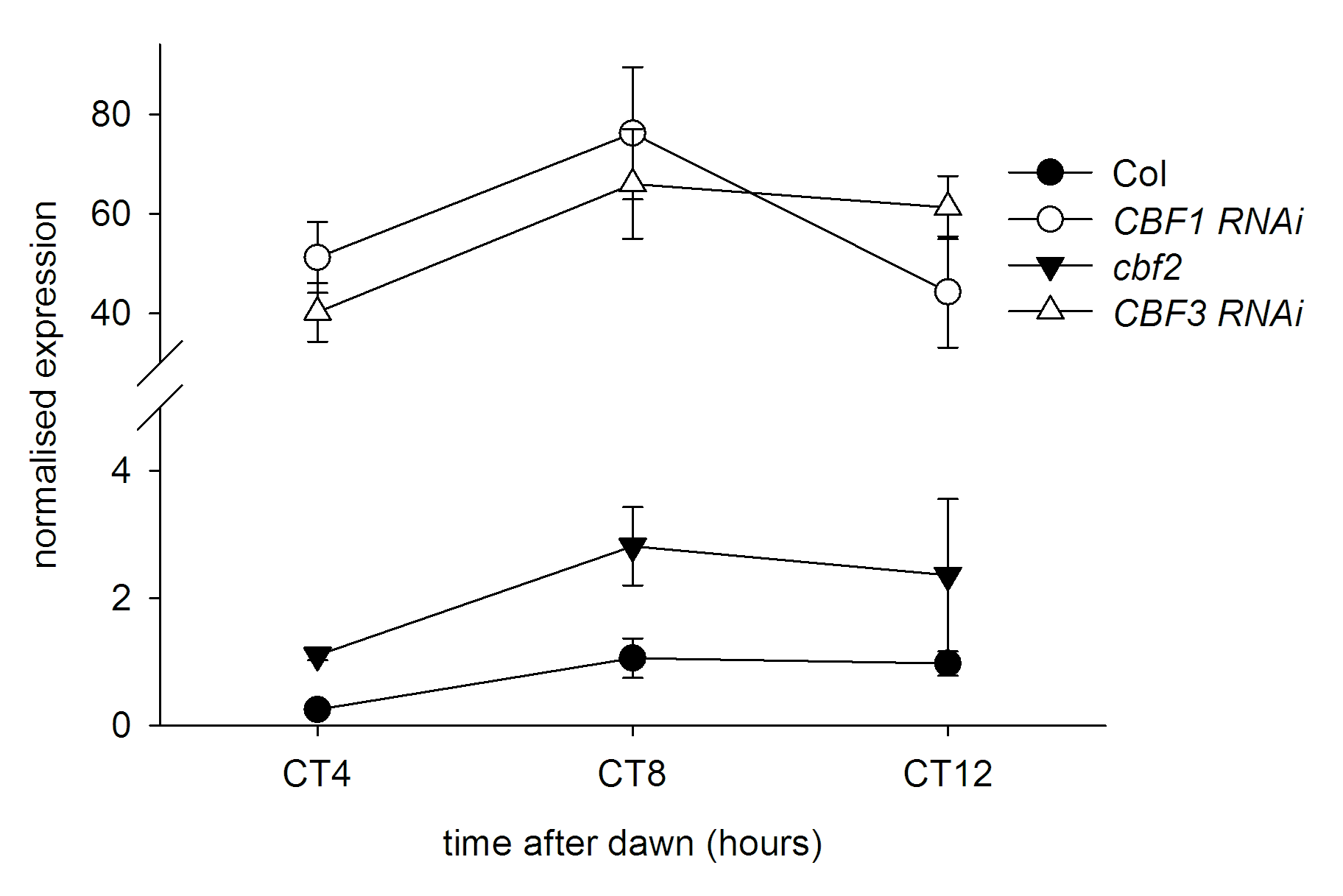
**

**Figure S4: Published CBF1 Real-time PCR primers appear to prime from *CBF1* and *CBF3*.** Chart to show *CBF1* expression (using primers published in Bienewska et al., 2008 and Dong et al., 2011) in wild type and *CBF1* RNAi, *cbf2* mutants and *CBF3* RNAi plants (Novillo et al., 2007). Both *CBF1* and *CBF3* primers detect elevated expression in *CBF3* RNAi plants.

CBF1_F

Primer ------------------------GGAGACAATGTTTGGGATGC---------------- 20

CBF1 CGAAGGTGCGTTTTATATGGATGAGGAGACAATGTTTGGGATGCCGACTTTGTTGGATAA 60

CBF3 CGAAAATGCGTTTTATATGCACGATGAGGCGATGTTTGAGATGCCGAGTTTGTTGGCTAA 60

***.*.*******.****

CBF1_R

Primer -------------------GGAGTTACTAATATTCG-ATAGTCG-------------- 24

CBF1 ---GGTGACGTGTCGCTTTGGAGTTACTAATATTCG-ATAGTCGTTTCCATTTTTGTA 54

CBF3 GATGACGACGTATCGTTATGGAGTTATTAAAACTCAGATTATTATTTCCATTTT---- 54

******* ***:* **. **: * .

.

**Figure S5: Alignment of published *CBF1*-specific primers with *CBF1* and *CBF3* cDNA sequences.** Although mis-matches occur, similarity is high and may require very specific PCR conditions to discriminate between the two transcripts. The CBF3 sequence shown is nucleotides 634-669 to 740-793 of the 908bp cDNA. The CBF3 RNAi construct reported by Novillo et al (2007) includes the 3’ end of CBF3 and starts at base number 730 and therefore is not reported to include the sequence complimentary to CBF1_F above.
