# Supplementary material for: Model selection reveals control of cold signalling by evening-phased components of the plant circadian clock
Source: Plant J. 2013 Aug 5;76(2):247–57. doi: 10.1111/tpj.12303 (PMC4278413; doi:10.1111/tpj.12303)
Supplement: Figure S1 — Generalised method used for comparing models. Figure S2. Effect of τ2 Figure S3. Effect of d. Figure S4. Published CBF1 Real-time PCR primers appear to prime from CBF1 and CBF3. Figure S5. Alignment of published CBF1-specific primers with CBF1 and CBF3 cDNA sequences. [file tpj0076-0247-SD2.docx]

**Supplementary Table 1.** Optimised new parameter values for each of the thirteen models.

**Supplementary Table 2: AICc^U^ analysis results.** Analysis was run with and without the penalising of the number of model parameters (2^nd^ term in (9)). *d* = 4.

**Supplementary Table 3.** Sensitivity heatmap fort the expression of *CBF3* at the indicated time after dawn. Values indicate the fold-change in *CBF3* mRNA value with respect 4 hour intervals where dawn is 0 and 24 hours. The 20 most sensitive parameters are shown, in addition to the parameters gC2, gC1 and gC4 which govern regulation of *CBF3* by *EC*, *TOC1* and *LHY*. This analysis shows that the model is robust to variation in new parameter values, with the exception of the *CBF3* mRNA degradation rate constant. Fast degradation is necessary to produce the sharp waveform observed. Increases in expression are shown in blue with –ve values, decreases in orange with +ve values.

**Supplementary Table 4.** Primer sequences for chromatin Immunoprecipitation. Positions for each primer pair are shown in Figure 5B.

**Figure S1: Generalised method used for comparing models.**  Models were fitted to data (red dashes, squares) with the resulting example simulation (blue, triangles) in 12 hrs light (12L):12 hrs dark (12D) cycles. $\sigma^{2}$ was calculated from the difference between the model simulations and datapoints at the specific times of the day. A prior model was constructed using a sinewave (black line). The difference between the models and prior model was calculated at the same timepoints as the models were compared to the data, $\left(M-\mu\right)^{2}$. The region around the prior model sinewave (grey dashes) represents the space in which a model may lie given a perturbation to the prior model parameters. This space is characterised by the uncertainty in parameter values, $\tau^{2}$.

**Figure S2: Effect of $\tau^{2}$.** The AICc^U^ analysis was carried out over a range of $\tau^{2}$ values (*d* = 4). Δ_s_ values for EC TOC1 D (black line) and EC D: LHY/CCA1 U (red line) show that there would be no change in which model was favoured over the range of $\tau^{2}$ values. If the lines crossed, then that would suggest that conclusions drawn from the analysis would change at a specific value of $\tau^{2}$.

**Figure S3: Effect of *d*. (a)** The AICc analysis was carried out over a range of *d* ≥ 2. When *d* < 3, EC D was more favoured than EC TOC1 D: LHY U. When *d* ≥ 3, EC TOC1 D: LHY U was the most probable model. d = repression/ downregulation; u = activation/ upregulation. **(b)** The difference between the penalty terms of EC TOC1 D: LHY U and EC D decreases as *d* is increased. This was calculated by taking the value of *Z* from (6) and subtracting (EC TOC1 D: LHY U – EC D).

**Figure S4: Published CBF1 Real-time PCR primers appear to prime from *CBF1* and *CBF3*.** Chart to show *CBF1* expression (using primers published in Bienewska et al., 2008 and Dong et al., 2011) in wild type and *CBF1* RNAi, *cbf2* mutants and *CBF3* RNAi plants (Novillo et al., 2007). Both *CBF1* and *CBF3* primers detect elevated expression in *CBF3* RNAi plants.

**Figure S5: Alignment of published *CBF1*-specific primers with *CBF1* and *CBF3* cDNA sequences.** Although mis-matches occur, similarity is high and may require very specific PCR conditions to discriminate between the two transcripts. The CBF3 sequence shown is nucleotides 634-669 to 740-793 of the 908bp cDNA. The CBF3 RNAi construct reported by Novillo et al (2007) includes the 3’ end of CBF3 and starts at base number 730 and therefore is not reported to include the sequence complimentary to CBF1_F above.
